# Uncovering a Latent Bioactive Interleukin‐6 Glycoform

**DOI:** 10.1002/anie.202411213

**Published:** 2024-10-24

**Authors:** Yanbo Liu, Yuta Maki, Ryo Okamoto, Ayano Satoh, Yasuto Todokoro, Yurie Kanemitsu, Keito Otani, Yasuhiro Kajihara

**Affiliations:** ^1^ Department of Chemistry Graduate School of Science Osaka University 1-1, Machikaneyama Toyonaka 560-0043 Japan; ^2^ Forefront Research Center Graduate School of Science Osaka University 1-1, Machikaneyama Toyonaka 560-0043 Japan; ^3^ Technical Support Division, Graduate School of Science Osaka University 1-1, Machikaneyama Toyonaka 560-0043 Japan; ^4^ Graduate School of Interdisciplinary Science and Engineering in Health Systems Okayama University 3-1-1, Tsushimanaka Okayama 700-0082 Japan.

**Keywords:** Glycoprotein, Protein folding chemistry, Protein aggregation, Protein synthesis, Interleukin 6

## Abstract

A bioinspired semisynthesis of human‐interleukin‐6 bearing N‐glycan at Asn143 (143glycosyl‐IL‐6) was performed by intentional glycosylation effects and protein folding chemistry for regioselective peptide‐backbone activation. 143Glycosyl‐IL‐6 is a genetically coded cytokine, but isolation was difficult owing to a tiny amount. IL6‐polypeptide (1–141‐position) with an intentionally inserted cysteine at 142‐position was expressed in *E. coli*. The expressed polypeptide was treated with a chemical folding process to make a specific helices bundle conformation through native two‐disulfide bonds (43–49 and 72–82). Utilizing the successfully formed free‐142‐cysteine, sequential conversions using cyanylation of 142‐cysteine, hydrazinolysis, and thioesterification created a long polypeptide (1–141)‐thioester. However, the resultant polypeptide‐thioester caused considerable aggregation owing to a highly hydrophobic peptide sequence. After the reduction of two‐disulfide bonds of polypeptide (1–141)‐thioester, an unprecedented hydrophilic N‐glycan tag was inserted at the resultant cysteine thiols. The N‐glycan tags greatly stabilized polypeptide‐thioester. The subsequent native chemical ligation and desulfurization successfully gave a whole 143glycosyl‐IL‐6 polypeptide (183‐amino acids). Removal of four N‐glycan tags and immediate one‐pot *in vitro* folding protocol efficiently produced the folded 143glycosyl‐IL‐6. The folded 143glycosyl‐IL‐6 exhibited potent cell proliferation activity. The combined studies with molecular dynamics simulation, semisynthesis, and bioassays predict the bioactive conformation of latent 143glycosyl‐IL‐6.

## Graphical abstract

Using intentional glycosylation effects and protein folding chemistry for regioselective peptide‐bacbone activation, a new bioinspired semisynthesis method can yield an undiscovered potent bioactive IL6‐derivative.

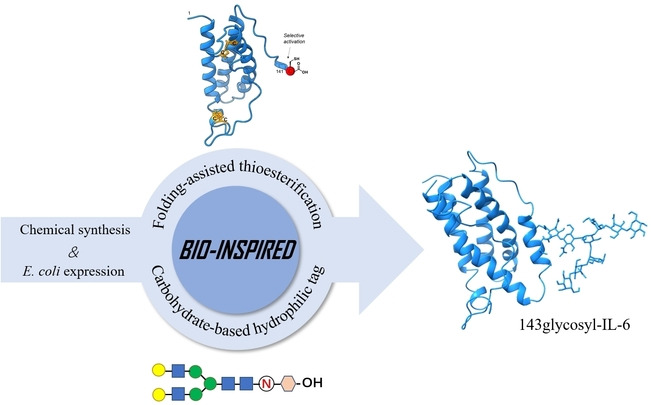

## Introduction

Natural proteins with post‐translational modifications (PTMs), show considerably diverse isoforms. As a ubiquitous PTM, N‐glycosylation gives diverse N‐glycosylation patterns (glycoform) and performs various important functions on glycoprotein.[^1^, ^2^] It is necessary to study the composition and behavior of each form of N‐glycans, even if they are present in small quantities in nature.

In recent years, highly sensitive mass spectrometry research has detected rare glycoforms that have not been isolated from natural sources before.[^3^, ^4^] N‐glycosylation occurs at the side chain of asparagine in the consensus sequence (Asn‐X‐Ser/Thr: X is any amino acid except for proline). However, almost all N‐glycosylation sites are not glycosylated simultaneously.^[5]^ Recent reviews discuss the emergence of active and inactive N‐glycosylation sites during the molecular revolution.[^6^, ^7^] However, the regulation of which consensus sequences are glycosylated has not been clearly understood, and the function of rare glycoforms remains unclear. Determining the specific N‐glycan structure after isolating rare glycoproteins which only trace amounts exist in our body such as cytokines from blood or natural sources is highly challenging. Unlike erythropoietin, which was isolated from urine samples totaling 2550 liters,^[8]^ native cytokines are typically found in serum at levels 0–5 pg/mL.^[9]^ This low concentration makes isolating and studying the individual N‐glycan structures and their positions in minor cytokines extreamly difficult. However, even small amounts of cytokines may potentially stimulate cells, highlighting the importance of studying these minor glycoforms. Therefore, in order to accelerate the study of glycan functions, chemical synthesis is essential for the preparation of such rare glycoforms of cytokines^[9]^ as well as understanding their biological activity.

**Figure 1:**
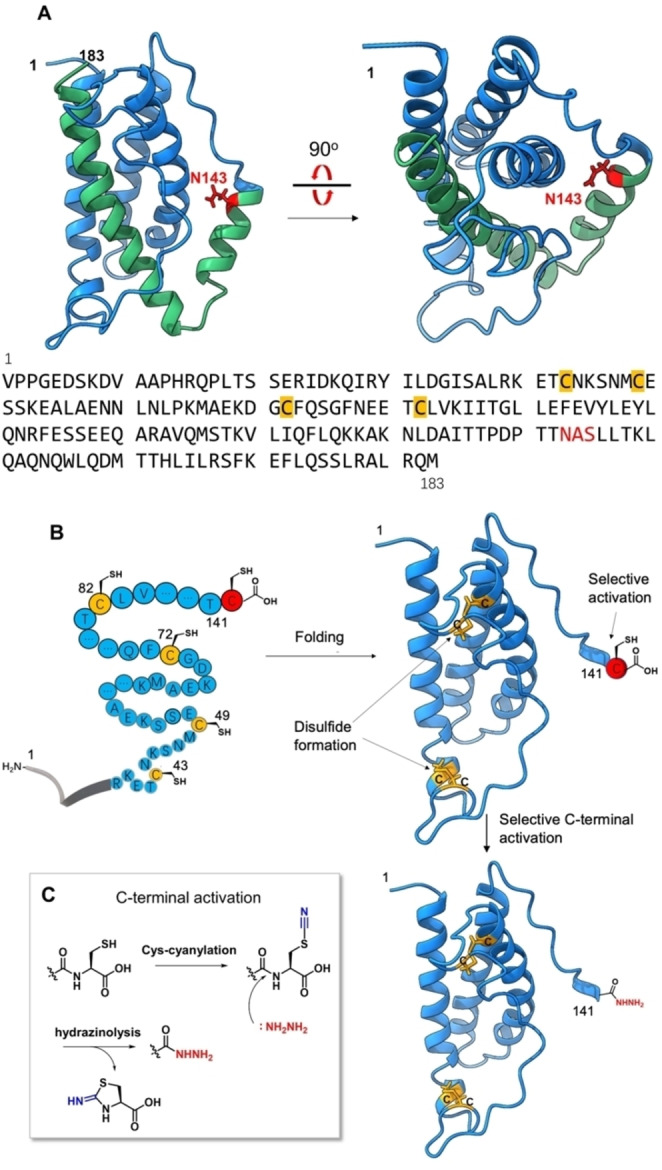
A unique protein structure of IL6 and a novel thioesterification by protein folding chemistry. **A**. Sequence, N‐glycosylation site (shown in red), and Cys forming disulfide bonds (shown in yellow) of human IL‐6. **B** The basic concept of folding‐assisted thioesterification. Selective C‐terminal Cys activation could be achieved after the folding process, which forms natural disulfide bonds to facilitate the formation of thermodynamically stable conformation. The folded structure (blue) was derived from PDB data (1IL6: Figure 1A) by the deletion of C‐terminal segment (142–183: Figure 1A dark green). **C**. C‐terminal Cys activation to produce peptide hydrazide as a thioester surrogate.

In this scenario, we selected human interleukin‐6 (IL‐6) bearing N‐glycosylation at Asn143 (143glycosyl‐IL‐6) as a synthetic target. Although the sequence of IL‐6 contains two consensus sequences for N‐glycosylation,^[10]^ only one of them (Asn44 glycosylated) is mainly found in recombinantly expressed IL‐6 and from natural sources.^[11]^ Semisyntheses of this glycoforms (44glycosyl‐IL‐6) were also performed by the sophisticated method.[^12^, ^13^] In addition, X‐ray crystallographic analysis (PDB: 1ALU, 1IL6) of IL‐6 showed an intriguing three‐dimensional structure around the 143 position (Figure 1A). Because it is known that glycans interfere with the growth of crystallization, X‐ray crystallographic analysis has usually been examined without glycans. Therefore, the current glycosylation site at the Asn143 area of IL‐6 in PDB data faces the interior of the IL‐6 protein. If the glycosylation occurs at the 143 position, these peptide areas with bulky glycan change their conformational properties and may face a bulk water layer in order to reduce steric hindrace of the glycan. Therefore, regarding a latent glycoform, the structure and bioactivity of 143glycosyl‐IL‐6 have not been investigated. Indeed, IL‐6 accelerates acute inflammation through the invasion of coronavirus (COVID‐19), but all functions of IL‐6 glycoforms are poorly understood.^[14]^

In the past decades, the chemical synthesis of homogeneous proteins with PTMs has been achieved by developing solid‐phase peptide synthesis (SPPS)^[15]^ and native chemical ligation (NCL).^[16]^ SPPS enables incorporating amino acid with PTMs.^[17]^

However, many synthetically difficult proteins with PTM remain to be synthesized, and these proteins with PTM show a highly hydrophobic character that seriously hinders even the chemical and semisynthesis.[^18^, ^19^] The target 143glycosyl‐IL‐6 also shows a highly hydrophobic character (Figure S1). Therefore, chemical and semisynthesis often causes considerable aggregation of peptide products, which is no longer useful for synthesis. On the other hand, biosynthetic pathways of such instable proteins are supported by chaperones for the protein folding and glycosylation for the stabilization of the resultant proteins.

To achieve efficient chemical synthesis, we studied a novel bioinspired method for selective chemical activation of the expressed long peptide and unprecedented efficient hydrophilic tag. Both methods were developed based on the intrinsic character of a naturally modified protein such as glycoproteins.

Regarding the first development in our bioinspired method, we used a protein folding chemistry to selectively activate the specific C‐terminus of peptide sequence to yield the thioester form of a long peptide (Figure 1 B). Protein folding is an intrinsic process to form thermodynamically stable conformation,^[20]^ facilitated by forming native disulfide bond patterns, which could serve as selective protection of native cysteine residues, resulting in a free cysteine intentionally installed (C‐terminal in this research). The free cysteine at the C‐terminal end can be used for thioesterification through cyanylation and subsequent hydrazinolysis.^[21]^ Additionally, the folded polypeptide exhibits better stability in solution due to the buried hydrophobic residues in the folded polypeptide molecule.^[22]^

Regarding the second advantage in our bioinspired method for the problem of hydrophobicity, we designed an unprecedented hydrophilic tag based on a human‐type glycan inspired by the native glycoprotein to improve its stability and solubility.[^23^, ^24^, ^25^] We found that the glycan‐based hydrophilic tag could dramatically improve stability by coating the hydrophobic region of peptides rather than conventional polylysine (polyLys)[^26^, ^27^, ^28^, ^29^, ^30^, ^31^] or polyarginine (polyArg) tags.[^26^, ^27^, ^32^, ^33^]

In this paper, we report a unique bioinspired semisynthetic strategy of an undiscovered bioactive 143glycosyl‐IL‐6 glycoform by protein folding–assisted thioesterification and N‐glycan‐hydrophilic tag.

## Results

### Synthetic Strategy by a Bioinspired Folding‐assisted Peptide‐thioesterification

First, we examined a semisynthetic strategy to synthesize 143glycosyl‐IL‐6 (Figure S1, S2). In this strategy, the sequence of IL‐6 was divided into five segments. However, owing to the terrible hydrophobic character, we gave up the segment condensation strategy in the presence of guanidine.

To solve these problems, we studied a novel expressed peptide thioesterification method inspired by protein folding to address the solubilization of hydrophobic IL6‐peptide and selective disulfide bond formation under thermodynamic control. During protein *in vivo* folding, hydrophobic residues are usually incorporated in hydrophobic interactions and hidden inside proteins,^[22]^ which produce soluble and bioactive proteins. Since the N‐terminal fragment of IL‐6 (1–141) contained the majority of the whole IL‐6 sequence and four cysteines that form native disulfide bonds (Figure 1 B), we supposed that the *in vitro* folding of the N‐terminal segment (1–141) could enable the selective activation of the intentionally added cysteine residue at desired C‐terminal position for thioesterification, namely, protein folding–assisted thioesterification. The Cys activation could be carried out by cyanylation of thiol on the cysteine side chain and then hydrazinolysis to produce a versatile peptidyl hydrazide^[34]^ as a thioester surrogate (Figure 1 C).^[21]^

**Figure 2:**
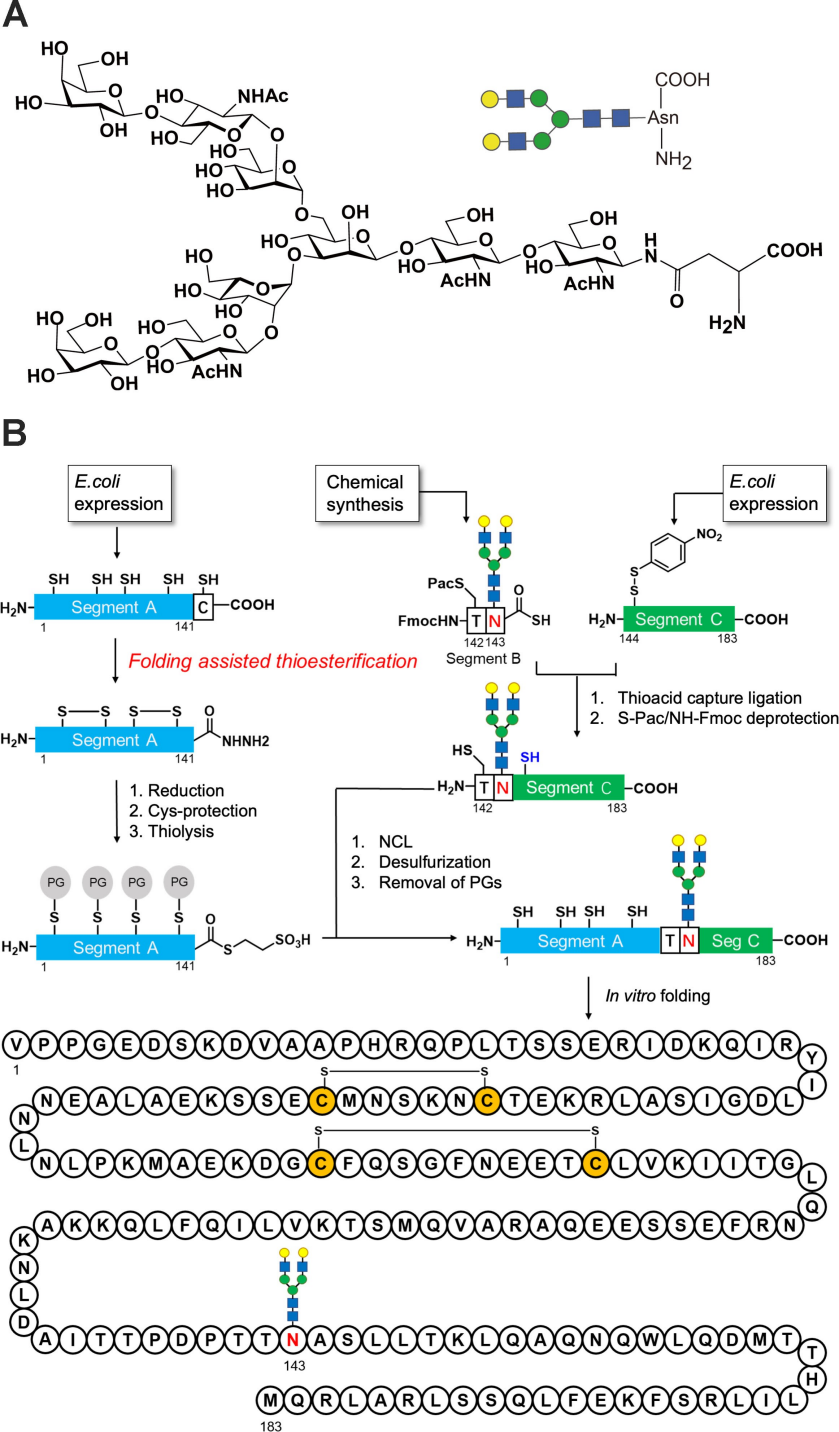
**A** glycan structure used in this synthesis. **B** A novel semisynthetic strategy for 143glycosyl‐IL‐6. Segments A (1–141) and C (144–183) were prepared by *E. coli* expression. For segment A, the selective activation of C‐terminal Cys residue toward thioester was carried out by folding‐assisted thioesterification. Glycopeptide segment B (142–143) was chemically synthesized.

Based on the protein folding–assisted thioesterification method, we designed a novel semisynthetic strategy toward 143glycosyl‐IL‐6 (Figure 2). In this strategy, we divided the sequence of IL‐6 into three segments: a long segment A (1–141) was designed to be prepared by *E. coli* expression and converted into a thioester derivative via protein folding–assisted thioesterification. Chemically synthesized glycopeptide segment B (142–143) and expressed peptide segment C (144–183) could be ligated using thioacid capture ligation.^[35]^ Finally, NCL could produce full‐length glycosylated IL‐6 for *in vitro* folding after desulfurization^[36]^ and removing protecting groups (PGs). As shown in Figure 2, this strategy does not include SPPS and may provide a substantial amount of 143glycosyl‐IL‐6 for crystallization or other applications. Regarding the N‐glycan structure, the native structures of IL‐6 are sialyl type and high‐mannose type N‐glycans.^[10]^ However, we selected asialo type glycan (Figure 2A) which was more feasible to manage than sialylglycan, because the synthesis of protein backbone was difficult due to hydrophobic character. Additionally, the first synthesis of glycosyl‐IL6 also employed asialo type glycan^[12]^.

### Folding‐assisted Peptide‐thioesterification

**Figure 3:**
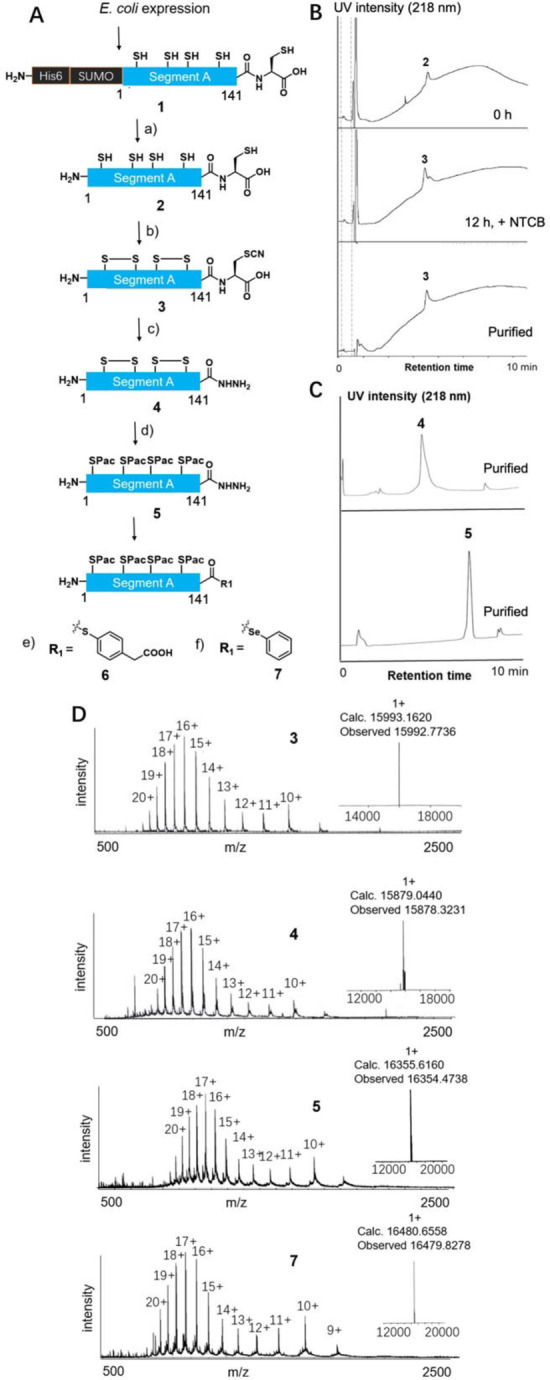
Synthesis results of segment A (1–141) hydrazide and thioester based on folding‐assisted thioesterification. **A**. Synthetic route. **B**. LC result of *in vitro* folding and cyanylation (0 h, 12 h + adding NTCB, purified). **C**. LC result of purified compounds **4** and **5** (purified). **D**. ESI‐MS of purified compounds **3**, **4**, **5**, and **7**. a) 50 mM Tris‐HCl, 75 m M NaCl, 1 mM DTT, pH 8.0, 30 °C, SUMO protease, 70 %. b) 1) 6 M Gu‐HCl, 0.2 M phosphate, air, pH 7.8, r.t. 2) 3 mM NTCB, 46 % for two steps. c) 1 M Gu‐HCl, 50 mM Tris‐HCl, 0.5 % v/v hydrazine, pH 10.7, 23 °C, 40 % yield. d) 1) 6 M Gu‐HCl, 0.2 M phosphate, 5 mM TCEP, pH 7.2; 2) 20 equiv. Pac‐Br in DMF, 50 % yield for two steps. e) 1) 6 M Gu‐HCl, 0.2 M phosphate, pH 3.0, 5 equiv. acetylacetone; 2) 200 mM MPAA, 65 % for two steps. f) 1) 6 M Gu‐HCl, 0.2 M phosphate, pH 3.0, 5 equiv. acetylacetone; 2) 50 mM diphenyl diselenide, 50 mM TCEP, 50 % for two steps.

First, we examined segment A's *E. coli* expression and thioesterification (1–141) in the folding assisted manner (Figure 1, 3). After *E. coli* expression and cleavage of His_6_‐small ubiquitin‐related modifier (SUMO) tag^[37]^ to produce compound **2**, we screened the conditions for *in vitro* folding of segment A (1–141), which is the key reaction in this method (Figure S3, S4, Table S1). Among several conditions we screened, the oxidative condition (air oxidation in 6 M Gu‐HCl, pH 7.8) exhibited good reaction yield^[38]^ and fewer byproducts compared with the dialysis method using red/ox reagents. In the latter method, we observed multiple byproducts, including misfolded peptide and red/ox reagent attachment to the C‐terminal cysteine residue.

After folding segment A (1–141), we examined the selective cyanylation at C‐terminal cysteine among five peptide backbone cysteine. The resultant folded segment A (1–141) was treated with 2‐nitro‐5‐thiocyanatobenzoic acid (NTCB) to give the folded segment A **3** with cyanylated Cys at C‐terminal (Figure 3B).^[39]^ This process could be performed in a one‐pot manner from polypeptide segment A (1–141) **2** in 46 % yield (2 steps).

Finally, the subsequent hydrazinolysis of segment A (1–141) and conversion into its peptide‐thioester was performed. Hydrazinolysis was successfully performed at pH 10.7, 0.5 % v/v hydrazine condition to afford peptidyl hydrazide **4** in 40 % yield as a thioester surrogate (Figure S7). If the cyanylation occurred at the internal cysteines of **2**, the longest peptide (1–141)‐hydrazine **4** could not be obtained. These data (Figure 3A, C, D, and Figure S7) indicated that disulfide bond formations under the folding condition occurred at the native positions and gave the free cysteine at the C‐terminal. In addition to these data, analysis of the circular dichroism spectrum of **3** (Figure S6) and its high mass data (Figure S5) suggested the formation of a helix bundle, indicating the correct folding product **3**. Because subsequent thioesterification and native chemical ligation require additive thiols, we reduced the disulfide bonds of **4** at this step. After the reduction of disulfide bonds of **4** with tris(2‐carboxyethyl)phosphine (TCEP), the protection of Cys residues with the phenacyl (Pac) group was performed (50 % yield, two steps).^[40]^ Then the resultant peptidyl hydrazide **5** was activated by acetylacetone to form a pyrazole intermediate, then converted into 4‐mercaptophenylacetic acid (MPAA) thioester **6** in 65 % yield.^[41]^ We also prepared a selenoester derivative **7** for chemical ligation.^[42]^ By utilizing folding‐assisted thioesterification, we could obtain a long peptide segment A containing 141 residues as a thioester form in only a few chemical conversion steps after *E. coli* expression.

### Synthesis of Glycosyl Polypeptide Segment BC (142–183) and Ligation

**Figure 4:**
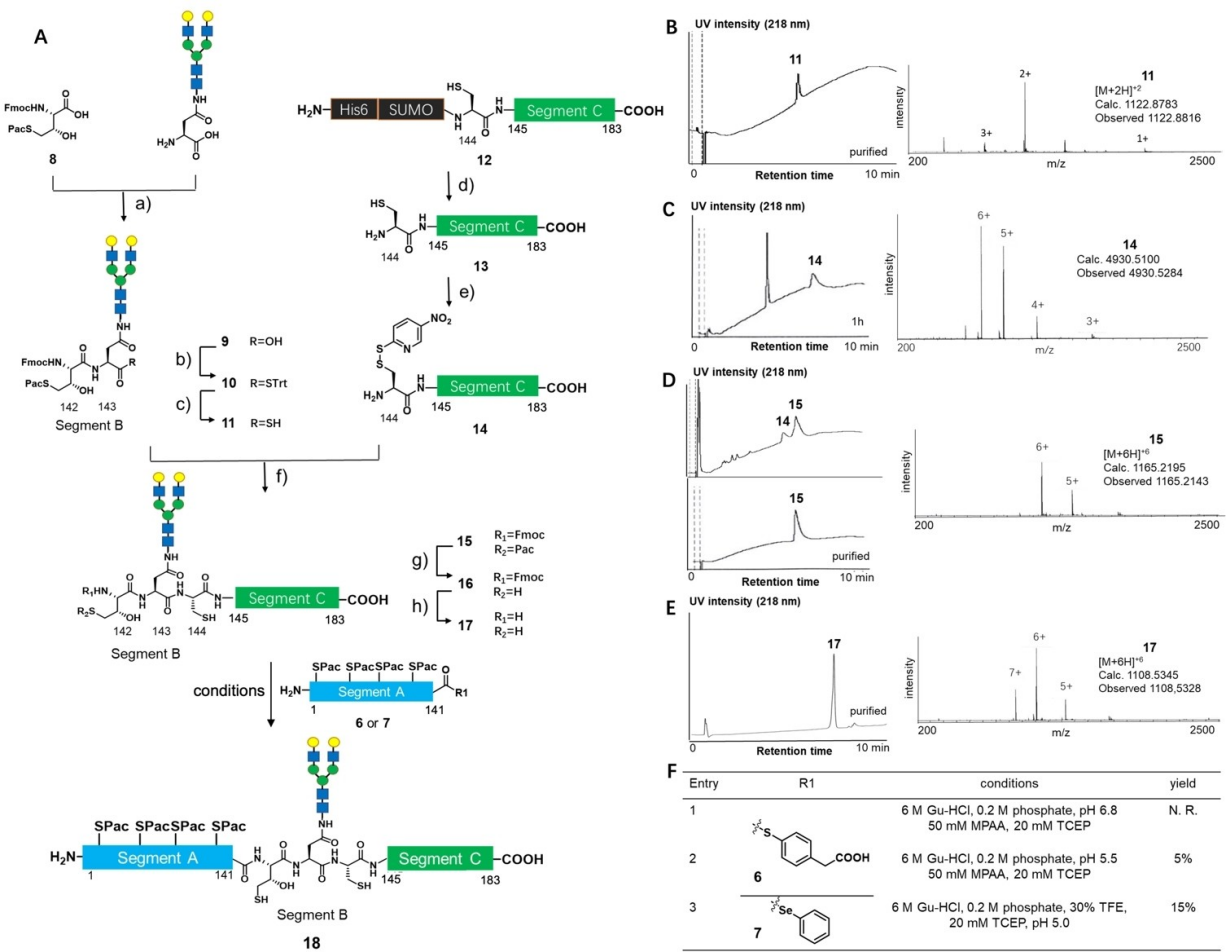
Synthesis results of glycopeptide segment BC (142–183) and ligation results segments A and BC. **A**. Synthetic route. **B**. LC–MS result of purified compound **11** (purified). **C**. LC–MS result of compound **14** (1 h). **D**. LC–MS result of thioacid capture ligation (1 h, purified). **E**. LC–MS result of purified compound **17**. **F**: Ligation results between **17** and either **6** or **7**. Both thioester and selenoester showed poor reaction yield. N.R.=no reaction. a) PyBOP, DIPEA, DMF/DMSO, 50 % yield. b) TrtSH, PyBOP, DIPEA, DMF, 0 °C, 45 % yield. c) 6 M Gu‐HCl, 0.2 M phosphate, 200 mM Na_2_S, pH 7, 88 % yield. d) 50 mM Tris‐HCl, 75 m M NaCl, 1 mM DTT, pH 8.0, 30 °C, SUMO protease, 60 % yield. e) 20 equiv. DTNP, 0.1 % TFA, 50 % v/v acetonitrile in H_2_O, 95 % yield. f) 6 M Gu‐HCl, 0.2 M phosphate, pH 5.6, 10 mM DTT for quenching, 80 % yield. g) Zn powder, 45 % v/v AcOH in 6 M Gu‐HCl, 0.2 M phosphate buffer, pH 4.8, 75 % yield. h) 20 % v/v piperidine in DMF, 81 % yield.

Next, we examined the synthesis of glycopeptide segment BC (142–183) by combining chemical synthesis and *E. coli* expression (Figure 4). Starting from L‐methionine methyl ester, γ‐mercapto Thr **8** was chemically synthesized based on the reported synthetic route,^[43]^ but the protecting group was modified (Figure S11 and S36). Asialo‐N‐glycosyl asparagine was prepared based on a semisynthetic protocol reported by our group.^[44]^ The amide coupling reaction between **8** and Asialo‐N‐glycosyl asparagine by PyBOP/DIEA activation produced N‐glycosyl dipeptide (segment B, 142–143 in 50 % yield) **9**, followed by conversion of its carboxylic acid to tritylthioester **10** in 45 %. Finally, thioester was converted to glycosyl dipeptide thioacid **11** in 88 % yield.^[45]^

The preparation of segment C (144–183) used an *E. coli* expression system and modification of the SUMO tag to improve its hydrophilicity.^[37]^ After expression, the His_6_‐SUMO tag was cleaved with SUMO protease to produce **13** in 60 % yield. Then, the Cys144 residue was modified with 5‐nitro‐2‐pyridyl disulfide to give peptide **14** in 95 % yield. The pyridyl disulfide group was used as a handle for ligation with peptidyl thioacid. The structure of **14** was confirmed by high‐resolution mass spectrometry (Figure S17).

Glycopeptide thioacid **11** and the C‐terminal peptide **14** were in hand; then, their coupling reaction was performed under the thioacid capture ligation (TCL).^[35]^ The TCL between **11** and **14** was carried out in 6 M Gu‐HCl buffer (pH 5.6) to produce segment BC **15** in 80 % yield after reduction of disulfide bond at 144 Cys with dithiothreitol (DTT) (Figure 4D). Finally, the Cys‐Pac and NH‐Fmoc PGs were removed by Zn/AcOH and piperidine/DMF to afford glycosyl polypeptide **17** in good yield.

Next, we examined the ligation between segment A thioester **6** and segment BC **17** to afford full‐length IL‐6 glycopolypeptide (1–183) **18** (Figure 4), however the product obtained in a low yield due to considerable peptide aggregation. The standard NCL condition under pH 6.8 failed to produce the ligated target **18**. The aggregation of segment A was indicated by the disappearance of signals in LC/MS at the very early stage in NCL. Lowering the pH to 5.5 slightly seemed to reduce aggregation and afforded target **18** in 5 % isolated yield. However, the yield could not be elevated. We supposed that the steric hindrance at the ligation junction (Thr‐Thr, flanked by N‐glycan), poor solubility, and considerable aggregation of segment A caused low yield. We optimized the reaction using a more reactive selenoester derivative **7** and adding 30 % v/v trifluoroethanol (TFE) as a cosolvent.^[46]^ Although the reaction was accelerated due to the remarkable reactivity of selenoester, we could only obtain ligated product **18** in 15 % isolated yield (Figure 4F, entry 3). We added 50 mM MPAA and 20 mM TCEP as additives in the thioester ligation reactions, but we didn't observe much thioester hydrolysis. Hydrolysis was not the main reason for the low yield (5 %). However, when we used selenoester **7** for ligation, we observed hydrolysis, which caused a low yield (15 %). Because we didn't observe S‐Pac deprotection at pH 5.5 (Figure 4F, entry 2), S‐Pac was stable under these conditions. We concluded that the low yields in ligation were due to aggregation. In addition, we found that the subsequent desulfurization of cysteine and S‐Pac deprotection showed poor yield due to peptide aggregation.

### Glycan‐based Hydrophilic Tag

**Figure 5:**
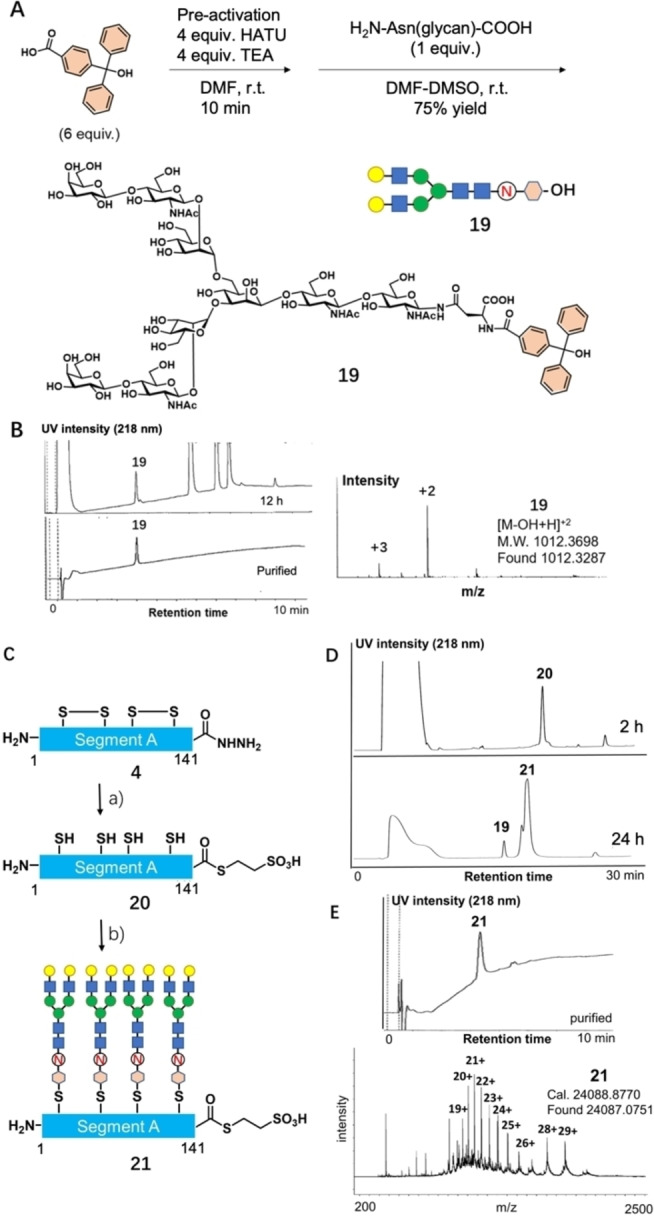
Synthesis of carbohydrate‐based hydrophilic tag using asialo complex‐type *N*‐glycan. **A**: Synthetic route. **B**: LC/ESI‐MS result of compound **19** (12 h, purified). Installation of hydrophilic tag on segment A (1–141). **C**: synthetic route. **D**: LC results of thioesterification and installation of hydrophilic tag. **E**: LC/ESI‐MS result of purified **21**. a) 1) 6 M Gu‐HCl, 0.2 M phosphate, pH 3.0, 10 equiv. NaNO_2_; 2) 200 mM MesNa, pH 6.8, 45 % for two steps. b) 16 equiv. HO‐trityl asialoglycosyl‐Asn **19**, HFIP, r.t. 80 % yield.

To solve the problem of hydrophobicity and improve ligation yield, we developed a novel hydrophilic tag based on glycan. In order to compare the efficiency of different hydrophilic tags, we prepared three kinds of hydrophilic tags using the trityl group^[26]^ as a scaffold for selective installation on Cys‐thiol of segment A (Figure 5). The conventional hydrophilic tag based on hexa‐Lys was synthesized based on a reported efficient method.^[26]^ Regarding carbohydrate‐based hydrophilic tag, we selected commercially available lactose as the second synthetic target (Figure S30). Since we established a large‐gram‐scale method to prepare complex‐type N‐glycan by isolating it from egg yolk,^[44]^ we have included asialo N‐glycan as an additional tag. These carbohydrate‐based hydrophilic tags were synthesized via an amide coupling reaction between 4‐(diphenylhydroxymethyl) benzoic acid and lactose amine/asialo N‐glycan by HATU/TEA activation to give compound **19** in 75 % yield (Figure 5).

With these hydrophilic tags in hand, we examined the installation of hydrophilic tags on segment A of IL‐6. From substrate **4**, we prepared segment A‐MesNa‐thioester **20** in 45 % yield (2 steps) as a substrate containing four free cysteines as modification sites (Cys43, 49, 72, and 82) (Figure 5). The installation reactions of hydrophilic tag were performed in 1,1,1,3,3,3‐hexafluoroisopropanol (HFIP).^[27]^ For the installation of the carbohydrate tag, we first examined the lactose tag, but the hydrophilicity was not improved (data not shown). Therefore, we focused on the N‐glycan tag **19**. After 48 h, LC/MS indicated that N‐glycan tags modified all four cysteines, and the substrate was converted to target **21** in 80 % yield (Figure 5). However, installing hexa‐Lys to segment A (1–141) **20** exhibited unexpected results (Figure S31). We only observed modification of two hexa‐Lys tags as a main product. The two modification positions with Lys‐tag were heterogeneous, and we observed increasing of two Lys tags by mass analysis (Figure S31). Installing the third and fourth hexa‐Lys tags no longer proceeded, even after extending reaction time or adding more tags. We hypothesized that the cationic characteristics of hexa‐Lys might impede the modification of the same cationic hexa‐Lys tag at a close position (Cys43–Cys49 and Cys72–Cys82) due to repelling force (Figure S32). Alternatively, a N‐glycan tag, which exhibits neutral characteristics as a natural product, could be installed more efficiently. HPLC analyses showed that four N‐glycan tags reduced hydrophobicity and aggregation of the resultant segment A **21** (Figure 5).

### Synthesis of Full‐length 143 glycosyl‐IL‐6

**Figure 6:**
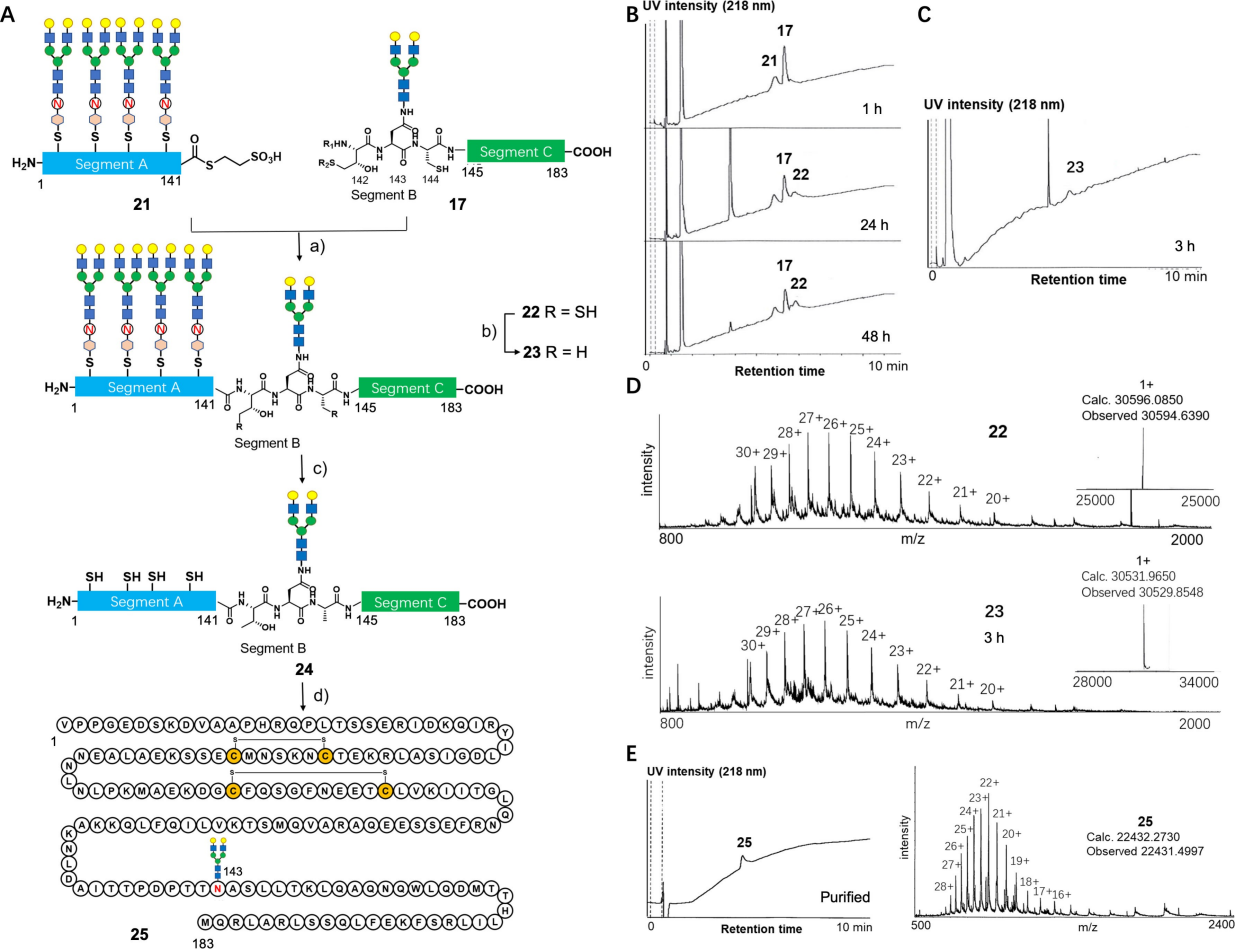
Synthesis results of ligation, desulfurization, removal of tag, and folding toward full‐length IL‐6. A. Synthetic route. B. LC result of native chemical ligation (1 h, 24 h, and 48 h). C. LC result of desulfurization (3 h). D. ESI–MS of purified compounds **22** and **23**. E. LC/ESI‐MS of folded glycoprotein IL‐6 **25**. a) 80 mM MPAA, 20 mM TCEP, 8 M Gu‐HCl, 0.2 M phosphate, pH 6.9, 48 h, 50 % yield (based on the consumption of **21**). b) 200 mM TCEP, 40 mM glutathione, 20 mM VA‐044, 6 M Gu‐HCl, 0.2 M phosphate, pH 6.9, 3 h, 78 % yield. c) 0.1 M HCl in HFIP. d) 5 % v/v HFIP, 1 mM cysteamine, 6 M Gu‐HCl, air, 50 mM Tris‐HCl, pH 7.5, 12 h, then dialysis, 33 % yield. The glycan tag **19** could be recovered in good yield in this process and was found to be used for the next glycan tag‐installation.

As we significantly improved the hydrophilicity of segment A, we repeated the NCL in the presence of hydrophilic N‐glycan tags in segment A (Figure 6A). This time, segment A's solubility was much improved, enabling us to perform NCL under 3 mM concentration of peptide substrates. In addition, the aggregation was remarkably reduced. Under 8 M Gu‐HCl buffer, MPAA/TCEP, pH 6.8 condition, we could obtain ligated product **22** in 50 % isolated yield after gel filtration (Figure 6B and D). Subsequently, desulfurization was performed using VA‐044/glutathione/TCEP to give the desulfurized product **23** in 78 % yield (Figure 6A and C).^[47]^ With glycan tags, all processes were efficiently performed.

The final steps included the removal of N‐glycan tags and the subsequent folding process, and this one‐pot manner successfully yielded the correctly folded 143glycosyl‐IL‐6 **25** (Figure 6A). The N‐glycan tags were removed before *in vitro* folding. Since the trityl group is labile under acidic conditions, substrate **23** was treated with 0.1 M HCl in HFIP for 30 min to produce **24** quantitatively. The reaction solution was directly used for *in vitro* folding by diluting HFIP solution with 6 M Gu‐HCl buffer (pH 8), which contained oxygen for air oxidation formation of disulfide bonds.^[38]^ After confirming the formation of disulfide bonds by LC/MS, rapid dialysis through a Tube‐O‐Dialyzer was performed to remove guanidine and produce folded 143glycosyl‐IL‐6 **25**. The final purification was performed with RP‐HPLC to yield the correctly folded 143glycosyl‐IL‐6 **25** in 33 % yield (Figure 6E: The yield was estimated by HPLC). The disulfide bond positions were also confirmed by peptidase digestion (Lysyl endopeptidase, Figure S29).

After RP‐HPLC purification, the folded 143glycosyl‐IL‐6 **25** could be estimated, but the solution was immediately used for bioassays due to instability. The fractions containing glycosyl IL‐6 eluted from RP‐HPLC were pooled for the biological assay. The estimation of 143glycosyl‐IL‐6 **25** in the solution was performed by Bradford estimation and a calibration curve on HPLC, and then the solution was diluted with a 1000‐fold buffer solution (phosphate‐buffered saline) for bioassays.

**Figure 7:**
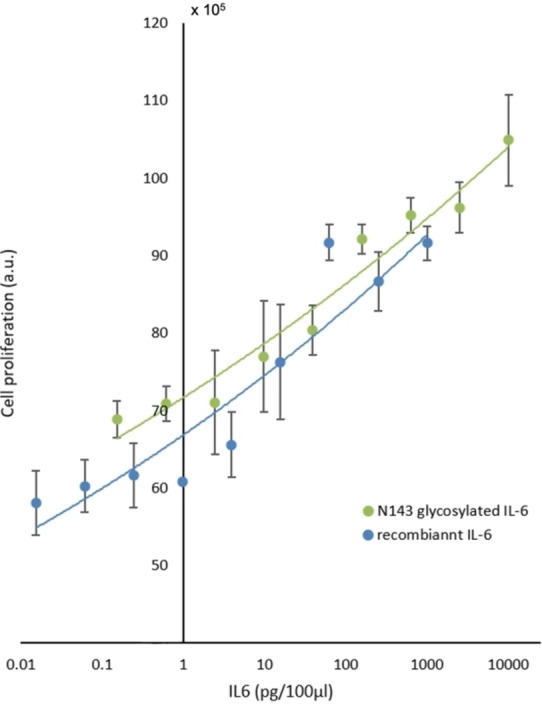
TF1 cell proliferation assay results. Green: synthetic Asn143 glycosylated IL‐6. Blue: commercially available recombinant hIL‐6 (without glycosylation). Horizontal axis: IL‐6 concentration (pg/100 μL). Vertical axis: cell proliferation (a.u.). The synthetic glycosylated IL‐6 exhibits comparable biological activity compared to nonglycosylated IL‐6. Error bars indicate the standard deviation of two or three biological replicates.

Finally, we examined the biological activity of 143glycosyl‐IL‐6 **25** by measuring the TF1 cell proliferation assay.^[48]^ Commercially available recombinant hIL‐6 without glycosylation was used as a control. The result (Figure 7) indicated that our synthetic 143glycosyl‐IL‐6 **25** exhibited efficient bioactivity compared to nonglycosylated IL‐6. 143glycosyl‐IL‐6 **25**, which has not been isolated from natural sources, exhibited cell proliferative activity with even femtogram order prepared by 1000‐fold dilution. 143Glycosyl‐IL‐6 **25** showed stability in buffer solution for a week, but its bioactivity gradually decreased.

## Discussion

In the past decade, several chemical methods for thioesterification of expressed peptides based on the activation of C‐terminal cysteine have been reported.[^21^, ^49^, ^50^, ^51^, ^52^] Along with these methods, protein folding‐assisted thioesterification described here, alternatively, is a new concept and takes advantage of only the intrinsic character of protein to achieve selective protection and selective activation of cysteine at the desired position. The folding for thioesterification succeeded due to several reasons: 1) All of the four native cysteines located on this partial sequence form native disulfide bonds. 2) This partial sequence is long enough to form several secondary structures (α‐helix). 3) The folding process facilitates the formation of a thermodynamically stable secondary structure of this partial sequence, which could bury hydrophobic residues inside the helix bundle. We suppose this method using a natural product character has the potential for the semisynthesis of chemically modified protein, but we need to develop it for making a general method.

In this research, we demonstrated a novel hydrophilic tag based on N‐glycan inspired by natural products, such as glycoproteins. Carbohydrates, especially N‐glycans, are widely found on natural proteins as ubiquitous PTM to improve their thermostability and solubility *in vivo*. Compared to polyLys/polyArg, carbohydrate‐containing multiple hydroxy groups exhibit a neutral character, which enables solubilizing effect under not only acidic conditions but also neutral and even basic conditions. In addition, N‐glycans exhibit bulky character due to their branched structure. We expect such bulky character could prevent aggregation on a hydrophobic peptide.^[24]^ Based on these considerations, we thought N‐glycans would be a better choice for hydrophilic tag than monosaccharides or disaccharides, which could not improve hydrophobicity (Figure S30). In order to evaluate hydrophobicity, 8‐anilino‐1‐naphthalenesulfonic acid (ANS) was used as a gauge.^[53]^ According to the result of the ANS fluorescent assay (Figure S33), the binding between ANS and the hydrophobic patch on the N‐terminal fragment was remarkably decreased after attaching glycan tags, which indicated that hydrophobic interaction could be suppressed.

After installing four N‐glycan tags, we examined NCL again. Due to better solubility and stability of the N‐terminal segment by the N‐glycan tags, the NCL yield was significantly improved to achieve 50 % yield due to higher peptide concentration (3 mM) and decreasing peptide aggregation. The better stability against aggregation also allowed us to extend the reaction time to 48 h. In order to avoid RP‐HPLC and lyophilization, which cause loss of peptide, we purified the reaction by using gel filtration[^12^, ^13^] to afford target peptide in 6 M Gu‐HCl buffer, which could be directly used for subsequent desulfurization.

Before the final protein folding step, the N‐glycan tag could be smoothly removed under HCl/HFIP and then the resultant solution could be directly used for *in vitro* folding after dilution with 6 M Gu‐HCl buffer. Since HFIP improves the formation of secondary structures such as α‐helices, we expect that 5 % HFIP could be helpful to improve folding efficiency as well as protein stability during the oxidative folding process. We added 1 mM cysteamine to facilitate the oxidative formation of disulfide bonds. This condition was inspired by the reported refolding condition of previous semi‐synthesis of IL‐6.[^12^, ^13^] In the case of folding assisted thioesterification, we did not add cysteamine in order to avoid disulfide bond formation between cysteamine and 142Cys‐thiol.

Regarding the final a one‐pot folding protocol, including removal of N‐glycan tags and *in vitro* folding, could afford folded glycoprotein **25** in good yield and avoid material loss during RP‐HPLC and lyophilization. It is worth noting that the hydrophilic N‐glycan tag **19** could be recovered under this condition for reuse (Figure S27).

Although IL‐6‐bearing N‐glycan at Asn143 was not isolated by recombinant expression[^54^, ^55^] or from a natural source^[10]^ due to a small amount, our result indicated that this glycoform exhibited comparable bioactivity to commercially available nonglycosylated IL‐6. Due to the low concentration of IL‐6 required for assessing its bioactivity, which is in the order of femtograms and prepared through a 1000‐fold dilution, it is difficult to draw a clear conclusion about the potency of the glycosylated and nonglycosylated forms. We could conclude that synthetic 143glycosyl‐IL‐6 **25** could employ a correctly folded form and show suitable biological activity. Because even a few molecules of a rare cytokine‐glycoform may efficiently give a signal to a single cell, we should not neglect the rare glycoforms and need to study the biological activity of individual glycoform through chemical synthesis.

According to the known structure of IL‐6 (PDB: 1ALU, 1IL6, 1P9 M) studied without N‐glycans, the residue Asn143 faces the inside of the IL‐6 structure. Modifying a bulky N‐glycan at this position should face the outside and change the conformation of the known IL‐6 structure. As a result, the N‐glycosylation at Asn143 may affect IL‐6 bioactivity and structure. So far, the structures of over 20,000 kinds of human proteins have been solved, but around 50 %–70 % of those human proteins are thought to be glycosylated.^[56]^ However, these structures of the individual glycoform have not been studied.

**Figure 8:**
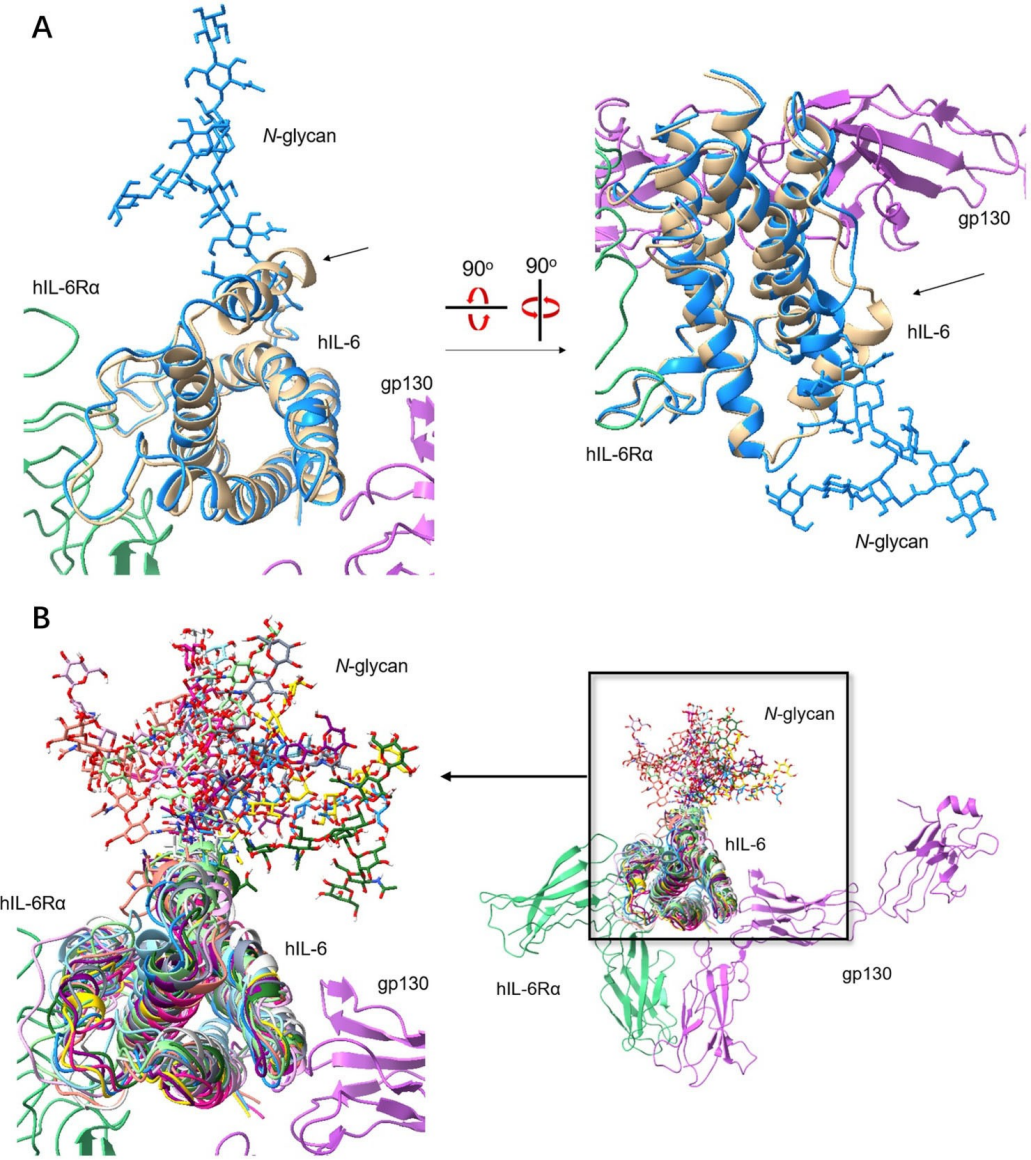
Conformational properties of a latent bioactive 143glycosyl‐IL6. A) Superimposed structure between IL‐6 complex (PDB: 1P9 M, IL‐6/IL‐6 receptor, gp130, and human IL‐6 receptor) and the predicted 143glycosyl‐IL‐6. Blue: predicted 143glycosyl‐IL‐6; gold: nonglycosylated IL‐6; green: human IL‐6 receptor; pink: gp130. The predicted 143glycosyl‐IL‐6 was determined through computational simulation and then superimposed with IL‐6/IL‐6 receptor complex (PDB: 1P9 M) by Chimera X. The arrow indicates different conformation of the helix containing Asn143 with and without N‐glycan. The other helices show consistent conformation with and without N‐glycan. B) The predicted form of 143glycosyl‐IL‐6 was used for molecular dynamics for 100 ns, and then the ten three‐dimensional structures formed were abstracted from the simulation at every 10 ns. Ten structures were used to make their superimposed structure. The resultant superimposed structure was inserted into the IL‐6/IL‐6 receptor complex (PDB: 1P9 M) by Chimera X. N‐glycan showed dynamic fluctuation, while the protein part exhibited a stable conformation which may not affect its binding with the IL‐6 receptor.

Recently a method combined with AlphaFold2 estimation and subsequent N‐glycosylation protocol^[57]^ was examined to evaluate unknown structures of glycoproteins. We also evaluated the conformational properties of 143glycosyl‐IL‐6 **25** by molecular dynamic simulations. First, we made a glycosylated structure by changing the helix angle around the Asn143 position (141–155 peptide) and adding N‐glycan at position 143. The resultant glycosyl form was minimized once and then used for molecular dynamics for 100 ns. The 10 conformers were abstracted at every 10 ns and used for the superimposed structure (Figure S35). The superimposed structure between our simulated one (Figure 8A: blue abstracted at 50 ns) and IL‐6 (gold color) with receptors (PDB: 1P9 M) showed a strong resemblance. However, only the short α‐helix containing glycosylation site Asn143 (indicated by arrow) showed remarkable conformational change: Asn143 of nonglycosylated IL‐6 faces the inside, while N‐glycosylated Asn143 residue faces outside due to steric hindrance of bulky N‐glycan. We considered that the flexibility of the adjacent loop might contribute to such conformational change. The results followed our hypothesis that glycosylated protein conformation might change compared to known structure in the PDB database, usually measured by X‐ray crystallography without glycans.

Figure 8B shows the superimposed structure of 10 conformers abstracted from 100 ns MD and IL‐6 receptors (PDB: 1P9 M). This superimposed structure suggested that the global conformation of protein parts did not change much, although N‐glycan exhibited considerable fluctuation. The helix bundle parts that interact with receptor hIL‐6Rα (green) and gp130 (pink) showed stable and suitable conformation for binding throughout the 100 ns MD simulation. The presence of N‐glycosylation at Asn143 does not interfere with the binding of IL‐6 to its receptor because the glycosylation site is opposite the binding interface between IL‐6 and receptors. This MD simulation result can explain why synthetic 143glycosyl‐IL‐6 exhibits comparable bioactivity to nonglycosylated IL‐6.

## Conclusions

We demonstrated a semisynthesis of 143glycosyl‐IL‐6, an undiscovered and highly hydrophobic protein. In this synthesis, we demonstrated a bioinspired strategy, such as glycoprotein form, in which N‐glycan is known to stabilize protein and protein folding to give thermodynamically stable protein conformation. Employing these bioinspired ideas, a very synthetically difficult glycoprotein, IL‐6, with an N‐glycan at the 143 position, could be efficiently synthesized without the SPPS protocol. Combining a new synthetic method, bioassay and MD simulation uncovered a latent potent bioactive glycoform. The current new semisynthetic method can be used for the synthesis of proteins with other PTMs to understand their undiscovered bioactive forms.

## Supporting Information

Supporting Information including additional Figures and Schemes, general experimental details, synthetic protocolsand characterization data are provided in a separate file.

## Conflict of Interests

The authors declare no conflict of interest.

1

## Supporting information

As a service to our authors and readers, this journal provides supporting information supplied by the authors. Such materials are peer reviewed and may be re‐organized for online delivery, but are not copy‐edited or typeset. Technical support issues arising from supporting information (other than missing files) should be addressed to the authors.

Supporting Information

## Data Availability

The data that support the findings of this study are available in the supplementary material of this article.
