# Supplementary material for: Uncovering a Latent Bioactive Interleukin‐6 Glycoform
Source: Angew Chem Int Ed Engl. 2024 Oct 24;63(50):e202411213. doi: 10.1002/anie.202411213 (PMC11609956; doi:10.1002/anie.202411213)
Supplement: Supplementary file 1 — Supporting Information [file ANIE-63-e202411213-s001.pdf]

Angewandte Chemie
Eine Zeitschrift der Gesellschaft Deutscher Chemiker

Supporting Information

Uncovering a Latent Bioactive Interleukin-6 Glycoform

*Y. Liu, Y. Maki, R. Okamoto, A. Satoh, Y. Todokoro, Y. Kanemitsu, K. Otani, Y. Kajihara**

Supporting Information

Uncovering a Latent Bioactive Interleukin-6 Glycoform

Yanbo Liu,[1] Yuta Maki,[1,2] Ryo Okamoto,[1,2] Ayano Satoh,[3] Yasuto Todokoro,[4] Yurie Kanemitsu,[1] Keito Otani,[1] Yasuhiro Kajihara[1,2]

[1]Department of Chemistry and [2]Forefront research center, [4]Graduate School of Science, Osaka University, Machikaneyama, Toyonaka, 560-0043 Japan

[3]Graduate School of Interdisciplinary Science and Engineering in Health Systems, Okayama University, 3-1-1 Tsushimanaka, Okayama 700-0082, Japan.

Contents

General method

Preparation of ultra-competent cell for transformation
Heat-shock transformation of plasmid
Cell lysis and denaturation of inclusion body
Ni-NTA His_6-tag affinity purification
SDS-PAGE electrophoresis (Laemmli method)

Figure S1. The failed first route for the synthesis of IL-6.
Figure S2. Hydrophobicity plot of IL-6.
Figure S3. Preparation of His6-SUMO-segment A (Val1-Thr141)-Cys **1**.
Figure S4. Preparation of segment A (Val1-Thr141)-Cys **2**.
Table S1. Optimization of folding condition for selective thioesterification.
Figure S5. Preparation of segment A -Cys(SCN) **3.**
Figure S6. Circular dichroism spectrum of folded segment A (1-141) **3**.
Figure S7. Preparation of segment A-hydrazide **4**.
Figure S8. Preparation of Pac protected segment A-$NHNH_2$ **5**.
Figure S9 Preparation of segment A-MPAA thioester **6**.
Figure S10. Preparation of segment A-SePh selenoester **7**.
Figure S11. Synthesis of S-Pac-(NH)-Fmoc-γ-mercapto-L-threonine **8**
Figure S12. Synthesis of glycopeptide **9** with γ-mercapto-threonine.
Figure S13. Synthesis of glycopeptide-thioester **10**.
Figure S14. Synthesis of glycopeptide-SH **11**.
Figure S15. Preparation of His6-SUMO-segment C (Cys144-Met183) **12**.
Figure S16. Preparation of segment C (Cys144-Met183) **13**.
Figure S17. Preparation of segment C [Cys144(S-Npys)-Met183] **14**.

Figure S18 Synthesis of segment BC [Thr142(S-Pac), Asn143 *N*-glycosylated] **15**.
Figure S19. Synthesis of segment BC [Thr142(SH)-Met183, Asn143 *N*-glycosylated] **16**.
Figure S20. Synthesis of segment BC [Thr142(SH)-Met183, Asn143 *N*-glycosylated] **17.**
Figure S21. Synthesis of Cys-Pac protected segment ABC**18**.
Figure S22. NH-Trityl-(N-asialo glycosyl)-asparagine **19**.
Figure S23. Synthesis of segment A [Val1-Thr141]-MesNa thioester **20**
Figure S24. Synthesis of segment A-MesNa **21** with four glycan tangs.
Figure S25. Synthesis of full length segment ABC **22** with four glycan tangs**.**
Figure S26. Desulfurization of full lemgth segment ABC **23** with four glycan tangs.
Figure S27. Synthesis of Segment ABC **24** and recovering glycan-tag **19**.
Figure S28. Folding of 143glycosyl-IL-6 **25**.
Figure S29. Disulfide bonds mapping of 143glycosyl-IL6 **25.**
Figure S30. Synthesis and installation of lactose-based hydrophilic tag.
Figure S31. Installation of Lys-tag to segment A.
Figure S32. Proposed mechanism for low installation yield of polyLys$_6$ tag.
Figure S33. Evaluation of hydrophobicity of peptide segments
Figure S34. Cell proliferation assays with synthetic 143glycosyl-IL6 **25**.
Figure S35. Superimposed structure of ten 143glycosyl-IL6.
Figure S36. ^{1}H NMR and ^{13}C NMR spectra of γ-mercapto threonine **8**.
Figure S37-40. Information of commercially available plasmid used for the prepration of segment A (Val1-Thr141)-Cys **2** and segment C (Cys144-Met183) **13**.

General method

All mass data are reported as high-resolution mass spectra (HRMS). All HRMS were reported on compact ESI Q-TOF (Bruker) mass spectrometry and Q Exactive Orbitrap LC-MS/MS System (Thermo Scientific). In terms of HRMS value, the peak with highest intensity was selected and written in the experimental section. LC-MS was analyzed on ultimate 3000 UHPLC (thermo scientific) - Amazon ETD (Bruker) or ultimate 3000 UHPLC (thermal scientific) – compact ESI Q-TOF (Bruker) using 0.1% HCOOH in ultrapure H_2O and 0.1% HCOOH, 90% acetonitrile (LC/MS grade, Fujifilm) in ultrapure H2O. A proteonavi C4 5 μm 2 mm I.D. x 50 mm (Shiseido) column or a cadenza CD-C18 (3 μm 50 x 2 mm, Imtakt) column was used for analysis. The eluent was monitored by UV-absorbance with 218 nm.

RP-HPLC purification was performed on Waters 2489 UV/visible detector and Waters 1525 binary HPLC pump using 0.1% TFA in diH_2O and 0.1% TFA in 90% acetonitrile. A proteonavi C4 (300 Å 5 μm, 250 mm x 10 mm, Shiseido) column or a proteonavi C4 (300 Å 5 μm, 250 mm x 4.6 mm, Shiseido) column was used. The eluent was monitored by UV-absorbance with 218 nm and 254 nm. Size-exclusive chromatography was performed on ultimate 3000 UHPLC (thermal scientific) using 6 M guanidine-HCl, 0.1 M phosphate in ultrapure H_2O (pH 6.5). A TSKgel SuperSW3000 (4.6 mm I.D. x 30 cm, 4 μm, TOSOH) was used for analysis. The eluent was monitored by UV-absorbance with 218 nm.

In order to estimate *E. coli* amount, OD600 value was measured on V-630 BIO spectrophotometer (JASCO) using LB media as blank control. The fed-batch high cell-density fermentation (Jar Fermenter) was performed using Bioneer Neo smart fermenter (B. E. Marubishi CO.). *E. coli* expression was performed using competent *E. coli* BL21(DE3, Nippon gene, Origami B (DE3, Novagen) and pET302/NT-His vector (invitrogen). The ultrapure water was produced by Simplicity UV water purification system (Merk) at 18.2 MΩ•cm resistivity (25°C). ANS fluorescence assay was measured on JASCO fp-6500 spectrofluorometer. CD spectra was measure on JASCO J-805 spectropolarimeter.

Plasmids of individual peptides were ordered to Thermo Fisher scientific according to the desired amino acid sequences.

Preparation of ultra-competent cell for transformation

The commercially available competent *E. coli* BL21 (DE3, Nippon gene) was used for preparation of ultra-competent *E. coli*. [H Inoue, *gene*, **1990**, 96, 23-8] The *E. coli* stock was streaked on LB plate (ampicillin -) and incubated at 37°C for 12 h. A single colony (1-3 mm diameter) was picked up and inoculated into 1ml SOB media (20 g/L tryptone, 5 g/L yeast extract, 0.5 g/L NaCl, 2.5 mM KCl, 10 mM $MgCl_2$, pH 7.0), which was then incubated at 25 °C, 150 rpm for 10-12 h. This 1 mL media was inoculated into 250 SOB media in 1 L flask, which was then incubated at 18 °C until OD600 reached 0.6-1.5. The 1 L flask was cooled down on ice for 10 min. The *E. coli* pellet was collected by centrifugation (4 °C, 3000 rpm, 15 min) then resuspended in 80 mL transformation buffer gently (via pipet-aid). The resuspended *E. coli* was put on ice for 10 min, then centrifuged (4 °C, 3000 rpm, 15 min) to collect *E. coli* pellet. The obtained pellet was resuspended in 20 mL transformation buffer in total, mixed gently using pipet-aid then treated with 7% v/v DMSO on ice for 10 min. The treated *E. coli* solution was dispensed into 1.5 mL-Eppendorf tube and chilled immediately

in liquid nitrogen, then stored in -80 °C.

Heat-shock transformation of plasmid

The "ultra" competent *E. coli* BL21 and pET302/NT-His vector was used for transformation. 100 μL of competent *E. coli* stock was mixed with 1 μL of plasmid in distilled H_2O and put on ice for 30 min. The heat-shock transformation was carried out by heating in 42 °C water bath for 90 s, then cooling down on ice for 2 min. LB media (10 g/L tryptone, 5 g/L yeast extract, 10 g/L NaCl, 200 μL, ampicillin -) was added and mixed. The resulting solution was incubated at 37 °C for 45 min to attain transformed recombinant *E. coli* for expression. The solution containing recombinant *E. coli* was inoculated on a prewarmed LB agar plate containing 100 μg/mL ampicillin (Unitech CO.) in 50/100/150 μL volume. After incubation at 37 °C for 12 h, a single colony was picked up for expression.

Cell lysis and denaturation of inclusion body

After cultivation, the *E. coli* pellet in LB media was collected by centrifugation under 4°C, 8000 rpm for 10 min. For 1 L media, 40 mL in total cell lysis buffer (50 mM Tris-HCl, 200 mM NaCl, 0.1% v/v Triton X, 5 mM $MgCl_2$, pH 7.5-8.0) was added to suspend *E. coli* pellet. The suspended *E. coli* in buffer was treated by sonication (on/off mode, 30s/30s x 4 rounds, amplitude 40) for cell lysis. After centrifugation (4°C, 8000 rpm, 10 min), supernatant was collected as a soluble part, while precipitation was collected as inclusion body. The denaturation of inclusion body was carried out using a buffer (50 mM phosphate, 300 mM NaCl, pH 8.0) containing Gn-HCl (6 M) as denaturant. For 1 L media, 40mL in total was added to suspend the inclusion body with mechanical assist. The suspended solution was centrifuged (4℃, 8000 rpm, 10 min) and supernatant was collected as denatured inclusion body. Both soluble part and inclusion body were purified with His_6-tag affinity purification.

Ni-NTA His_6-tag affinity purification

The His_6-tag affinity purification was carried out using complete His-Tag purification resin (Roche). The ethanol for preserving Ni-NTA resin was eluted, followed with distilled H_2O elution (5 x resin volume) for washing. The "equilibrium" step was carried out by elution of either lysis buffer (pH 7.5-8) or 6 M Gn-HCl buffer (pH 8) in 5 x resin volume. The sample solution containing peptide for purification was loaded for three times. 6 M Gn-HCl buffer as a "washing buffer" was eluted by gravity (5 x resin volume) to remove low affinity peptide in a column. Imidazole (500 mM in 6 M Gn-HCl buffer) as a "elution buffer" was used for elution by gravity (5 x resin volume) for pooling target peptide in fractions. Finally, the Ni-NTA resin was washed with distilled H_2O and preserved in ethanol under 4 °C.

SDS-PAGE electrophoresis (Laemmli method)

Sample solution (5 μL) was mixed with 1.0 M DTT (1 μL), 5 x sample buffer (250 mM Tris-HCl, 8% SDS, 40% glycerol, 0.1% bromophenol blue, 4 μL) and distilled H_2O (10 μL). The mixture was heated at 95 °C for 5 min by thermal cycler (2720 thermal cycler, applied biosystems) for complete denaturation of sample. The gel (SuperSep Ace, 15%, 17 well,

wako) was set into the apparatus and 1 x running buffer (25 mM Tris-HCl, 191 mM glycine, 0.1% SDS, pH 8.3) was added into reservoir. Pretreated sample solution (18 µL) as well as marker (5 µL, Precision Plus Protein, WesternC Standards, 161-0376, BIO-RAD) were loaded into the well. The electrophoresis was performed under 25 mA and 100 V condition until BPB reached the bottom. The gel was washed with diH_2O for three times, stained with CBB Stain One Super (Nacalai Tesque) at 25 ^oC, 50 rpm for 30 min, then destained with kimwiper in distilled H_2O at 25 ^oC, 50 rpm for 10 h.

The failed first semi-synthetic strategy

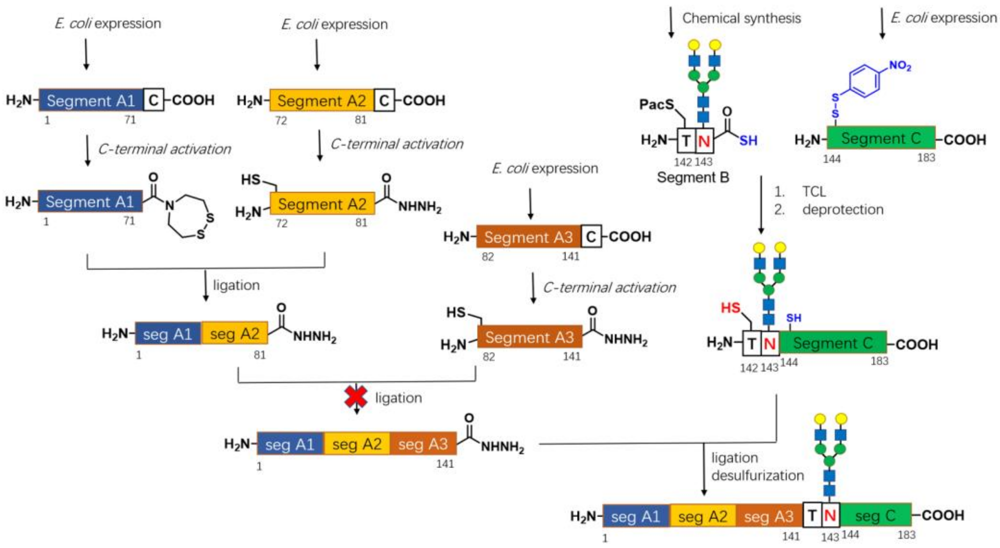

Figure S1. The first route for the synthesis of IL-6 by means of segment condensation strategy.

In the previous semi-synthetic strategy, we divided IL-6 sequence into five segments. Segment A1 (1-71), segment A2 (72-81), segment A3 (82-141) were prepared by *E. coli* expression and converted to corresponding thioester surrogate by using expressed peptide thioesterification methods. Segment B containing γ-mercapto Thr (142) and asialo N-glycosyl-Asn (143) could be chemically synthesized. Segment C (144-183) is prepared by *E. coli* expression. In terms of expressed peptide thioesterification, for segment A1, we activate C-terminal Cys via *N-S* acyl shift under acidic condition to produce oxidated bis-mercaptoethylamine (BSEA) amide derivative as thioester surrogate. [Ryo Okamoto et al., *J. Org. Chem.*, **2022**, 87, 114-124] For segment A2 and A3, we activate C-terminal Cys by S-cyanylation and hydrazinolysis to produce peptidyl hydrazide. These thioesterification methods for expressed peptide based on chemical activation of C-terminal Cys residue are developed in our group. However, due to the remarkable hydrophobicity of segment A3 (82-141) which caused poor solubility and irreversible aggregation, the subsequent reactions including expressed peptide thioesterification and NCL with segment A1A2

showed low yield. We failed to obtain segment A1A2A3 (1-141) via this synthetic route.

Hydrophobicity plot of IL-6 (Kyte & Doolittle method)

The hydrophobicity plot of IL-6 sequences was predicted based on Kyte-Doolittle scale. A window size of 9 was used. (*J. Mol. Biol.*, **1982**, 157:105-132)

As shown in the result, three parts of IL-6: Ser20-Lys40, Glu80-Leu125, and Pro140-Met180 exhibit considerable hydrophobicity. The blue square indicates the N-terminal peptide (1-141) prepared by expression and used for folding assisted thioesterification.

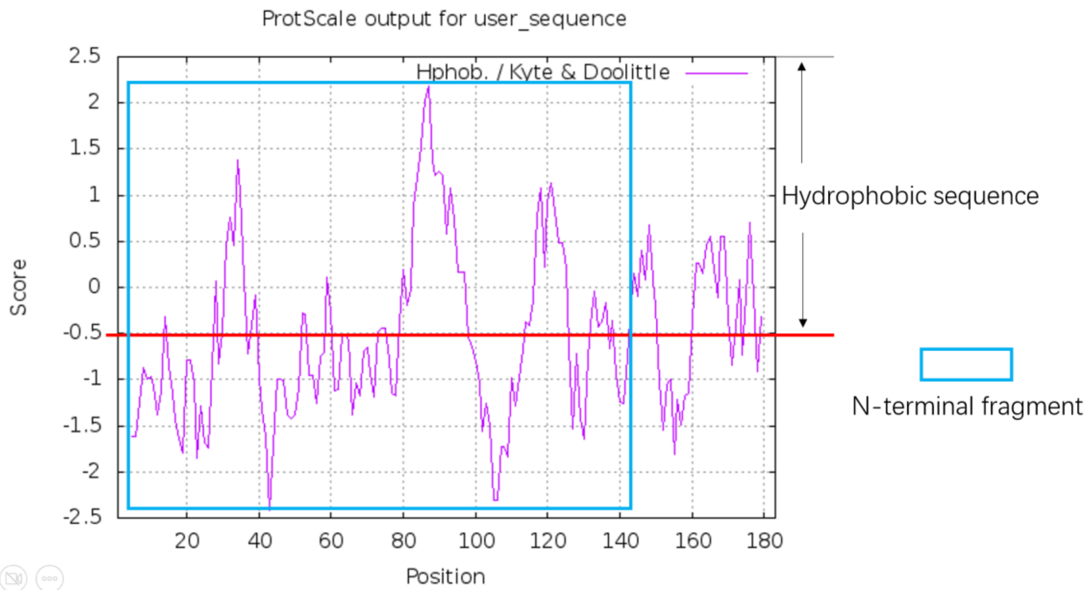

Figure S2. Hydrophobicity plot of IL-6.

E. coli expression of His6-SUMO-segment A (Val1-Thr141)-Cys 1

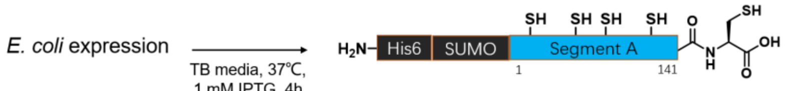

1

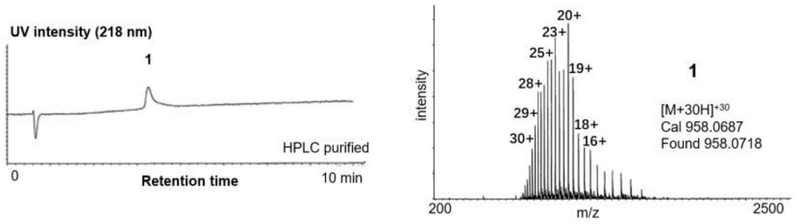

Figure S3. Preparation of His6-SUMO-segment A (Val1-Thr141)-Cys **1**.

The competent *Escherichia coli* strain Origami B (DE3, Novagen) and pET302/NT-His vector (invitrogen^{TM}) encoded recombinant peptide were used for heat-shock transformation to afford recombinant *E. coli*, which is incubated on LB agar plate containing 100 µg/mL ampicillin (Unitech CO.) at 37 ºC for 12 h. A single colony was picked up and inoculated into 10ml LB media containing 100 µg/mL ampicillin and incubated at 37℃ for 12 h as the starter culture. The starter culture was inoculated at 1:100 dilution into 1 L TB media containing 100 µg/mL ampicillin and incubated at 37 ºC until OD600 reached 0.6-1.0 (3-4 h). Isopropyl β-D-thiogalactopyranoside (IPTG) was added in 1 mM final concentration to start overexpression and the 1 L media was further incubated at 37℃ for 4 h. The 1 L expression media was centrifuged to collect *E. coli* pellet for cell lysis. After cell lysis with a cell lysis buffer (50 mM Tris-HCl, 200 mM NaCl, 0.1% v/v Triton X, 5 mM $MgCl_2$, pH 7.5-8.0), only inclusion body (precipitation) was collected by centrifugation. The inclusion body was treated with 6M Gu-HCl buffer (pH 8.0) for denaturation. Denatured inclusion body was purified by Ni-NTA His6 tag affinity purification then purified with RP-HPLC purification (proteonavi C4 300Å 5 µm, 250 mm x 10 mm, Shiseido, 0.1% TFA: 0.1% TFA in 90% MeCN= 70/30 to 10/90 over 60 min at 2.5 mL/min) to afford recombinant peptide **1** in 30 mg/L yield after lyophilization. ESI-MS: m/z calculated for $C_{1245}H_{1996}N_{352}O_{400}S_{13}$: 958.0687 $[M+30H]^{+30}$, observed 958.0718

701.2797 $[M+41H]^{+41}$, 718.7774 $[M+40H]^{+40}$, 737.1986 $[M+39H]^{+39}$, 756.5640 $[M+38H]^{+38}$, 776.9849 $[M+37H]^{+37}$, 798.5451 $[M+36H]^{+36}$, 821.3285 $[M+35H]^{+35}$, 845.4551 $[M+34H]^{+34}$, 871.0424 $[M+33H]^{+33}$, 898.2300 $[M+32H]^{+32}$, 927.17780 $[M+31H]^{+31}$, 958.0718 $[M+30H]^{+30}$, 991.0496 $[M+29H]^{+29}$, 1026.4082 $[M+28H]^{+28}$, 1064.3867 $[M+27H]^{+27}$, 1105.2882 $[M+26H]^{+26}$, 1149.4783 $[M+25H]^{+25}$, 1197.3183 $[M+24H]^{+24}$, 1249.3349 $[M+23H]^{+23}$, 1306.0874 $[M+22H]^{+22}$, 1368.2236 $[M+21H]^{+21}$, 1436.6081 $[M+20H]^{+20}$

SUMO cleavage: Synthesis of segment A (Val1-Thr141)-Cys 2

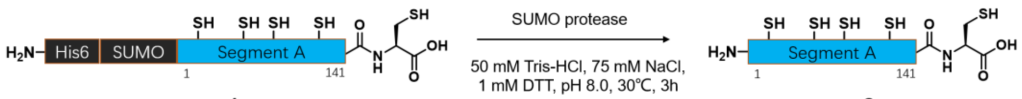

1 **2**

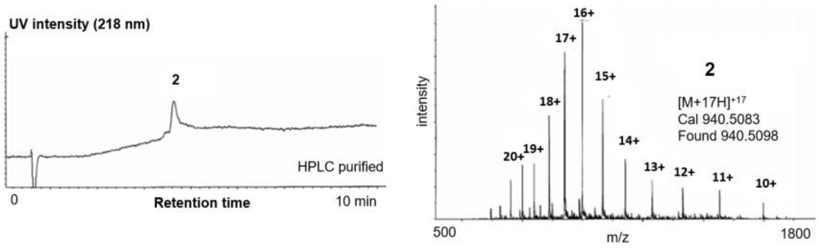

Figure S4. Preparation of segment A (Val1-Thr141)-Cys **2**.

To SUMO cleavage buffer (50 mM Tris-HCl, pH 8.0, 10 mL) containing NaCl (75 mM) and 1,4-Dithiothreitol (DTT, 1 mM) was dissolved His_6-SUMO-segment A (Val1-Thr141)-Cys **1** (5 mg, 0.17 μmol). A stock solution containing a large excess amount of recombinant SUMO protease was added. The reaction solution was shaken at 30ºC for 3 h until the reaction completion was monitored with LC-MS. The segment A in the reaction solution was purified with RP-HPLC (proteonavi C4 300Å 5 μm, 250 mm x 10 mm, Shiseido, 0.1% TFA: 0.1% TFA in 90% MeCN= 60/40 to 10/90 over 60 min at 2.5 mL/min) to afford segment A (Val1-Thr141)-Cys **2** (1.95 mg, white solid) in 70% yield after lyophilization. ESI-MS: m/z calculated for $C_{693}H_{1122}N_{190}O_{225}S_8$: 1000.5373 $[M+16H]^{+16}$, observed 1000.5405 726.9619 $[M+22H]^{+22}$, 761.5351 $[M+21H]^{+21}$, 799.5590 $[M+20H]^{+20}$, 841.5883 $[M+19H]^{+19}$, 888.2915 $[M+18H]^{+18}$, 940.5098 $[M+17H]^{+17}$, 999.2010 $[M+16H]^{+16}$, 1065.7510 $[M+15H]^{+15}$, 1141.8050 $[M+14H]^{+14}$, 1229.5741 $[M+13H]^{+13}$, 1331.9580 $[M+12H]^{+12}$, 1452.9595 $[M+11H]^{+11}$, 1598.1642 $[M+10H]^{+10}$, 1775.6357 $[M+9H]^{+9}$

Screening of segment A (1-141) peptidyl cysteine folding conditions

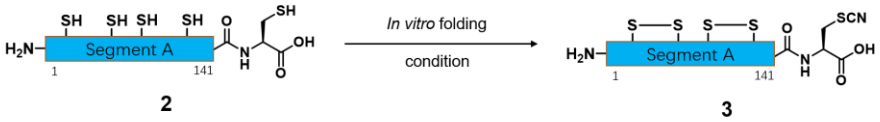

The in vitro folding condition of segment A (1-141) peptidyl cysteine was screened as follow. We examined stepwise dialysis as well as oxidative folding via air oxidation or adding oxidants. The results suggested that stepwise dialysis under red/ox reagent condition (cysteine/cystine) resulted in side-reactions, which was mainly due to attachment of cysteinyl disulfide bond on C-terminal cysteine. The air oxidation in 6.0 M guanidine buffer under slightly basic condition (pH 7.8-7.9) smoothly produced the folded segment A (1-141) without such side reactions. The air oxidation condition is also compatible with one-pot cyanylation by using NTCB. Although DMSO oxidation produced folded peptide faster than air oxidation, this condition is not compatible with subsequent one-pot cyanylation. Oxidation via oxidant including DTNP or DPDS lead to undesired byproduct due to attachment of disulfide bond on C-terminal cysteine. Based on these results, we suppose that air oxidation at pH 7.8 is the best condition for in vitro folding of segment A (1-141).

Table S1. Optimization of folding condition for selective thioesterification.

entry	condition	result
1	Stepwise dialysis with cysteine/cystine	side reactions

2	Air oxidation, 6.0 M Gu-HCl, 0.2 M phosphate, pH 7.8-7.9, 12 h	55% yield
3	Air oxidation, 6.0 M Gu-HCl, 0.2 M phosphate, pH 7.3, 12 h	30% yield
4	30% DMSO, 6.0 M Gu-HCl, 0.2 M phosphate, 2h	61% yield
5	DTNP, 6.0 M Gu-HCl, 0.2 M phosphate, pH 7, 10 min	side reactions
6	DPDS, 6.0 M Gu-HCl, 0.2 M phosphate, pH 7, 10 min	side reactions

Oxidative folding and cyanylation: Synthesis of segment A (Val1-Thr141, Cys43-S-S-Cys49, Cys72-S-S-Cys82)-Cys(SCN) 3

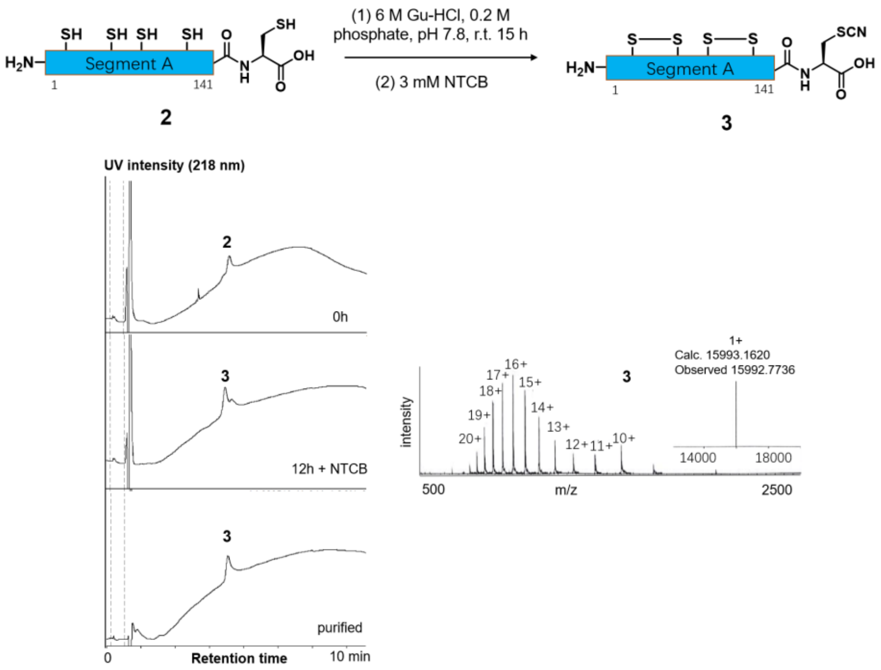

Figure S5. Preparation of segment A (Val1-Thr141, Cys43-S-S-Cys49, Cys72-S-S-Cys82)-Cys(SCN) **3.**

The segment A (Val1-Thr141)-Cys **2** (1.95 mg, 0.12 μmol) was dissolved in buffer solution (0.2 M phosphate, pH 7.2, 10 mL) containing Gn-HCl (6 M) and the final pH value was adjusted to 7.8 with 5 M NaOH aq. The solution was vortexed and shaken at 23°C until the completion of the reaction monitored by LC-MS. 2-Nitro-5-thiocyanatobenzoic acid (3 mM in a reaction mixture) was added to the reaction mixture and the mixture was shaken for 10 min at room temperature. The yellow color indicated that the reaction occurred. The product in the reaction mixture was purified by RP-HPLC (proteonavi C4 300Å 5 μm, 250 mm x 10 mm, Shiseido, 0.1% TFA: 0.1% TFA in 90% MeCN= 60/40 to 10/90 over 60 min

at 2.5 mL/min) to afford segment A (Val1-Thr141, Cys43-S-S-Cys49, Cys72-S-S-Cys82)-Cys(SCN) **3** (0.9 mg, white solid) in 46% yield for two steps after lyophilization. ESI-MS: m/z calculated for $C_{694}H_{1117}N_{191}O_{225}S_8$: 15993.1620 $[M+1H]^{+1}$, observed 15992.7736 (deconvoluted)

800.5983 $[M+20H]^{+20}$, 842.6734 $[M+19H]^{+19}$, 889.4434 $[M+18H]^{+18}$, 941.7095 $[M+17H]^{+17}$, 1000.4973 $[M+16H]^{+16}$, 1067.1373 $[M+15H]^{+15}$, 1143.2926 $[M+14H]^{+14}$, 1231.1711 $[M+13H]^{+13}$, 1333.6846 $[M+12H]^{+12}$, 1454.8421 $[M+11H]^{+11}$, 1600.2305 $[M+10H]^{+10}$, 1777.8332 $[M+9H]^{+9}$

CD spectrum of folded segment A (1-141) **3**

The substrate was dissolved in a buffer solution (50 mM Tris-HCl, pH 8.0) to 100 μg/mL concentration and added in cylindrical quartz cell (10 mm ID x 1 mm). The CD spectra was measured on JASCO J-805 spectropolarimeter. Parameter: response 0.5 seconds, wavelength 265-185 nm, resolution 0.2 nm, scan speed 50 nm/min.

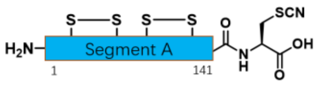

3

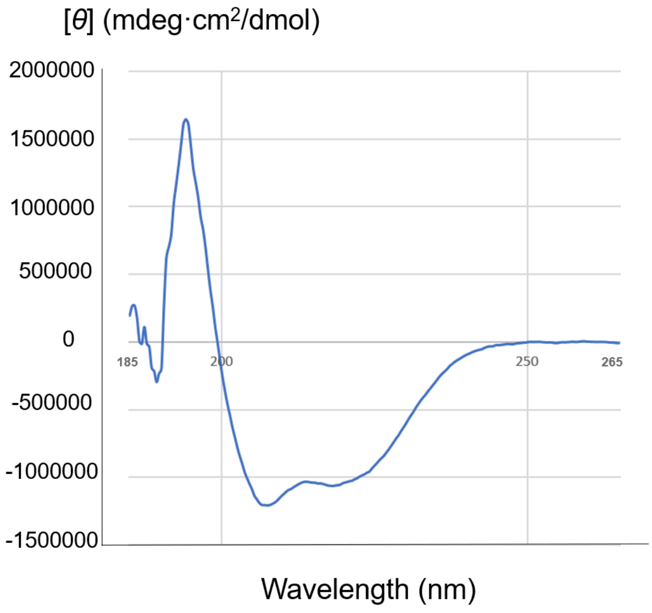

Figure S6. Circular dichroism spectrum of folded segment A (1-141) **3**.

Hydrazinolysis: Synthesis of segment A (Val1-Thr141, Cys43-S-S-Cys49, Cys72-S-S-Cys82)-NHNH$_2$ 4

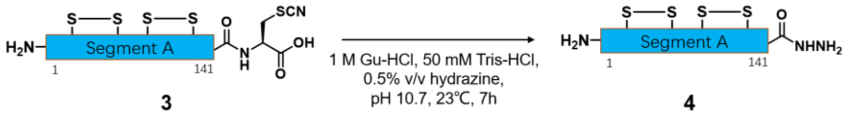

3 **4**

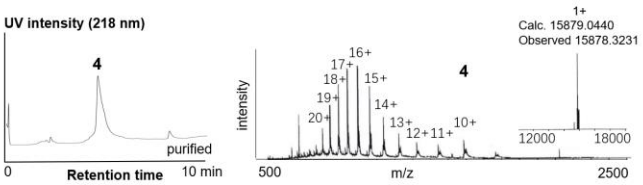

Figure S7. Preparation of segment A (Val1-Thr141, Cys43-S-S-Cys49, Cys72-S-S-Cys82)-hydrazide **4**.

To a buffer solution (50 mM Tris-HCl, pH 9.0, 8 mL) containing Gn-HCl (1.0 M) was added 0.5% v/v hydrazine monohydrate. The pH value was adjusted to 10.7 with 5.0 M NaOH aq. Compound **3** (1.5 mg,0.10 μmol) was dissolved and the solution was shaken at 23 °C for 7 h until the completion of reaction monitored by LC-MS. The reaction mixture was purified with RP-HPLC (proteonavi C4 300Å 5 µm, 250 mm x 4.6 mm, Shiseido, 0.1% TFA: 0.1% TFA in 90% MeCN= 70/30 to 20/80 over 30 min at 1.0 mL/min) to give segment A (Val1-Thr141, Cys43-S-S-Cys49, Cys72-S-S-Cys82)-$NHNH_2$ **4** (0.65 mg, white solid) in 40% yield after lyophilization. ESI-MS: m/z calculated for $C_{690}H_{1115}N_{191}O_{223}S_7$: 15879.0440 $[M+1H]^{+1}$, observed 15878.3231 (deconvoluted)
794.8987 $[M+20H]^{+20}$, 836.6851 $[M+19H]^{+19}$, 883.0914 $[M+18H]^{+18}$, 934.9892 $[M+17H]^{+17}$, 993.3623 $[M+16H]^{+16}$, 1059.4737 $[M+15H]^{+15}$, 1135.1324 $[M+14H]^{+14}$, 1222.4531 $[M+13H]^{+13}$, 1324.0898 $[M+12H]^{+12}$, 1444.4797 $[M+11H]^{+11}$, 1588.8028 $[M+10H]^{+10}$

Pac protection: Synthesis of segment A [Val1-Thr141, Cys43(SPac), Cys49(SPac), Cys72(SPac), Cys82(SPac)]-$NHNH_2$ 5

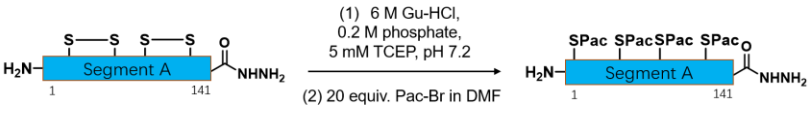

4 **5**

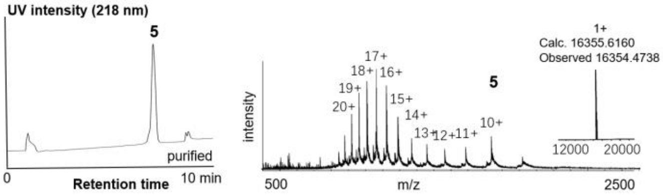

Figure S8. Preparation of segment A [Val1-Thr141, Cys43(SPac), Cys49(SPac), Cys72(SPac), Cys82(SPac)]-$NHNH_2$ **5**.

To a buffer solution (0.2 M phosphate, pH 7.2, 3 mL) containing Gn-HCl (6 M) and segment A (Val1-Thr141, Cys43-S-S-Cys49, Cys72-S-S-Cys82)-$NHNH_2$ **4** (0.65 mg, 0.04 μmol) was added TCEP (3 mM in a reaction mixture). The pH value was adjusted to 7.2 and the reaction mixture was shaken at room temperature for 20 min. After the completion of reaction monitored by LC-MS, DMF (20 μL) containing phenacyl bromide (0.13 mg, 0.64 μmol) was added for Pac protection. The reaction mixture was shaken at room temperature for 1-2 h and monitored by LC-MS. The product in the mixture was purified by RP-HPLC (proteonavi C4 300Å 5 µm, 250 mm x 4.6 mm, Shiseido, 0.1% TFA: 0.1% TFA in 90%

MeCN= 70/30 to 20/80 over 30 min at 1.0 mL/min) to give segment A [Val1-Thr141, Cys43(SPac), Cys49(SPac), Cys72(SPac), Cys82(SPac)]-NHNH$_2$ **5** (0.35 mg, white solid) in 50% yield for two steps after lyophilization. ESI-MS: m/z calculated for $C_{722}H_{1143}N_{191}O_{227}S_7$: 963.0783 $[M+17H]^{+17}$, observed 963.0796 (deconvoluted)
779.8278 $[M+21H]^{+21}$, 818.7481 $[M+20H]^{+20}$, 861.8096 $[M+19H]^{+19}$, 909.6263 $[M+18H]^{+18}$, 963.0796 $[M+17H]^{+17}$, 1023.1894 $[M+16H]^{+16}$, 1091.3403 $[M+15H]^{+15}$, 1169.2277 $[M+14H]^{+14}$, 1259.0892 $[M+13H]^{+13}$

Synthesis of segment A [Val1-Thr141, Cys43(SPac), Cys49(SPac), Cys72(SPac), Cys82(SPac)]-MPAA thioester 6

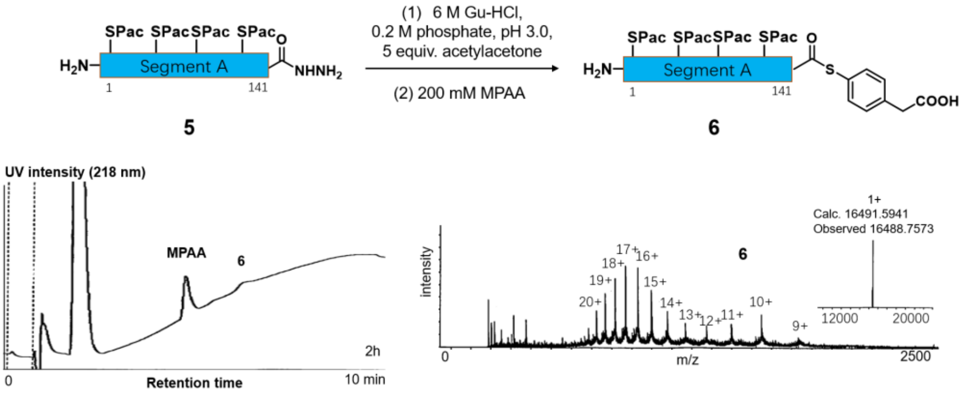

Figure S9 Preparation of segment A [Val1-Thr141, Cys43(SPac), Cys49(SPac), Cys72(SPac), Cys82(SPac)]-MPAA thioester **6**.

To a buffer solution (0.2 M phosphate, pH 3.0, 31 μL) containing Gn-HCl (6 M) and segment A [Val1-Thr141, Cys43(SPac), Cys49(SPac), Cys72(SPac), Cys82(SPac)]-NHNH$_2$ **5** (0.5 mg, 0.03 μmol) was added acetylacetone (5 mM in a reaction mixture). The reaction was shaken at room temperature and monitored by LC-MS. After completion of reaction, a buffer solution (0.2 M phosphate, pH 3.0, 31 μL) containing Gn-HCl (6 M) and MPAA (1.04 mg, 6.2 μmol) was added. The reaction was shaken at room temperature and monitored by LC-MS. Purification with RP-HPLC (proteonavi C4 300Å 5 μm, 250 mm x 4.6 mm, Shiseido, 0.1% TFA: 0.1% TFA in 90% MeCN= 70/30 to 20/80 over 30 min at 1.0 mL/min) afford segment A [Val1-Thr141, Cys43(SPac), Cys49(SPac), Cys72(SPac), Cys82(SPac)]-MPAA thioester **6** in 65% yield for two steps after lyophilization. ESI-MS: m/z calculated for $C_{730}H_{1147}N_{189}O_{229}S_8$ 971.0775 $[M+17H]^{+17}$, observed 971.0690
786.3319 $[M+21H]^{+21}$, 825.5974 $[M+20H]^{+20}$, 868.9917 $[M+19H]^{+19}$, 917.1716 $[M+18H]^{+18}$, 971.0690 $[M+17H]^{+17}$, 1031.6816 $[M+16H]^{+16}$, 1100.5097 $[M+15H]^{+15}$, 1178.9353 $[M+14H]^{+14}$, 1269.4504 $[M+13H]^{+13}$, 1375.1561 $[M+12H]^{+12}$, 1500.1714 $[M+11H]^{+11}$, 1650.0817 $[M+10H]^{+10}$

Synthesis of segment A [Val1-Thr141, Cys43(SPac), Cys49(SPac), Cys72(SPac), Cys82(SPac)]-SePh selenoester 7

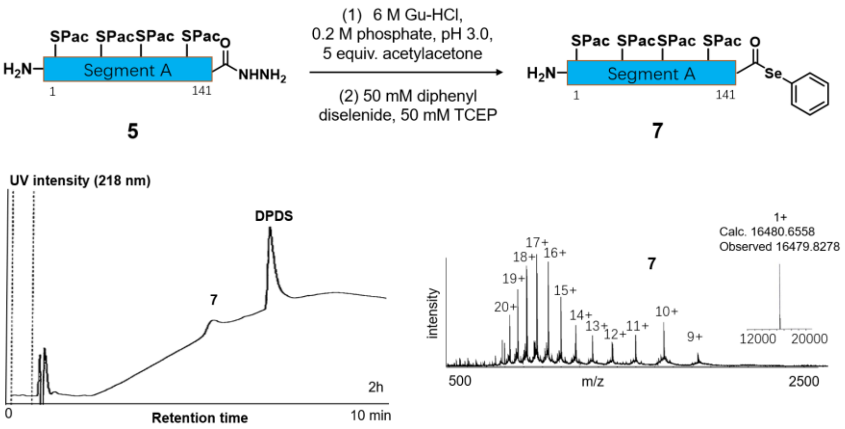

Figure S10. Preparation of segment A [Val1-Thr141, Cys43(SPac), Cys49(SPac), Cys72(SPac), Cys82(SPac)]-SePh selenoester **7**.

To a buffer solution (0.2 M phosphate, pH 3.0, 62 μL) containing Gn-HCl (6 M) and segment A [Val1-Thr141, Cys43(SPac), Cys49(SPac), Cys72(SPac), Cys82(SPac)]-$NHNH_2$ **5** (1.0 mg, 0.06 μmol) was added acetylacetone (5 mM in a reaction mixture). The reaction was shaken at room temperature and monitored by LC-MS. Diphenyl diselenide (1.9 mg, 6.2 μmol) and TCEP (1.7 mg, 6.2 μmol) was dissolved in buffer solution (0.2 M phosphate, pH 3.0, 62 μL) containing Gn-HCl (6 M) and pretreated with sonication for 20 min until yellow solid dissolved. The sonicated solution of diphenyl diselenide and TCEP was quickly added into reaction solution. The reaction mixture was shaken at room temperature until reaction completion was observed in LC-MS. RP-HPLC purification (proteonavi C4 300Å 5 μm, 250 mm x 4.6 mm, Shiseido, 0.1% TFA: 0.1% TFA in 90% MeCN= 70/30 to 20/80 over 30 min at 1.0 mL/min) afforded segment A [Val1-Thr141, Cys43(SPac), Cys49(SPac), Cys72(SPac), Cys82(SPac)]-SePh selenoester **7** in 50% yield for two steps after lyophilization. ESI-MS: m/z calculated for $C_{728}H_{1145}N_{189}O_{227}S_7Se$ 970.4269 $[M+17H]^{+17}$,observed 970.4312. 785.7983 $[M+21H]^{+21}$, 825.0379 $[M+20H]^{+20}$, 868.3953 $[M+19H]^{+19}$, 916.5972 $[M+18H]^{+18}$, 970.4312 $[M+17H]^{+17}$, 1031.0538 $[M+16H]^{+16}$, 1099.7169 $[M+15H]^{+15}$, 1178.1989 $[M+14H]^{+14}$, 1268.7493 $[M+13H]^{+13}$, 1374.4048 $[M+12H]^{+12}$, 1499.1842 $[M+11H]^{+11}$, 1649.0034 $[M+10H]^{+10}$

Segment B: Synthesis of (NH)-Fmoc-γ-mercapto-L-threonine 8

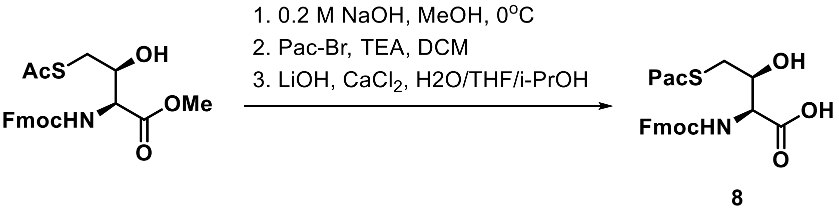

Figure S11. Synthesis of S-Pac-(NH)-Fmoc-γ-mercapto-L-threonine **8.**

S-acetyl-(NH)-Fmoc-γ-mercapto-L-threonine methyl ester was synthesized according to reported synthetic protocol [Chen J. et al. *tetrahedron* **2010**, 66, 2277-2283].

For the synthesis of compound **8**: To a MeOH solution (5.9 ml) containing S-acetyl-(NH)-Fmoc-γ-mercapto-L-threonine methyl ester (170 mg, 0.396 mmol) was added 0.2 M NaOH aq (3.9 ml). The reaction mixture was stirred at 0°C for 20min. 1 M HCl aq. was added to quench the reaction. The solution was diluted with EtOAc, washed with 1 M HCl aq. and brine, and the organic phase was dried with $MgSO_4$. After concentration, the residual was dried in vacuo and dissolved in DCM (8 ml). Phenacyl bromide (79 mg, 0.396 mmol) and triethylamine (60 μl, 0.435 mmol) was added, and the reaction mixture was stirred at room temperature. The reaction was monitored by TLC. After finishing of the reaction, the mixture was washed with 1 M HCl aq. and brine, dried with $MgSO_4$, and concentrated. The residual was dissolved in THF (6.4 ml) and iPrOH (1.6 ml) solution. A water solution (1.6 ml) containing LiOH (38 mg, 1.584 mmol) and $CaCl_2{\cdot}2H_2O$ (873 mg, 5.940 mmol) was added, and the mixture was stirred at room temperature. The reaction was monitored by TLC. After finishing, the solution was diluted with water, and extracted with Et_2O twice. The pH value of water phase was adjusted to 1.0 via 1% v/v HCl aq., then extracted with EtOAc twice. The EtOAc layer was combined, concentrated and purified with silica column purification (DCM:MeOH=10:1) to produce (NH)-Fmoc-γ-mercapto-L-threonine **8** in 44% yield as a white solid.

^{1}H NMR (400 MHz, CD_3OD) δ: 7.90 (2H, d, J = 7.5 Hz, phenacyl), 7.67 (2H, d, J = 7.2 Hz, Fmoc), 7.56 (1H, t, J = 7.6 Hz, 7.7 Hz, phenacyl), 7.50 (2H, t, J = 7.6 Hz, phenacyl), 7.40 (2H, mm, Fmoc), 7.26 (2H, dd, J = 6.4 Hz, 7.5 Hz, Fmoc), 7.20 (2H, dd, J = 6.8 Hz, 7.4 Hz, Fmoc), 4.27 (1H, m, Fmoc), 4.23 (1H, d, J = 3.2 Hz, Fmoc), 4.19 (1H, d, J = 3.2 Hz, Fmoc), 4.12 (1H, d, J = 7.3 Hz, αH), 4.00 (2H, dd, J = 7.1 Hz, phenacyl), 3.90 (1H, m, βH), 2.88 (1H, dd, J = 2.8 Hz, 5.6 Hz, γH), 2.55 (1H, dd, J = 5.8 Hz, 7.4 Hz, γH)

^{13}C NMR (400 MHz, CD_3OD) δ: 195.19, 159.11, 157.54, 141.17, 135.32, 133.24, 129.30, 128.46, 128.42, 127.38, 126.79, 124.89, 119.51, 78.41, 70.35, 66.74, 57.63, 37.18, 35.00

ESI-MS: m/z calculated for $C_{27}H_{25}NO_6S$ 492.1475 $[M+H]^{+1}$, observed 492.1229

Segment B: Synthesis of γ-mercapto-L-threonyl-(N-asialo-glycosyl) asparagine 9

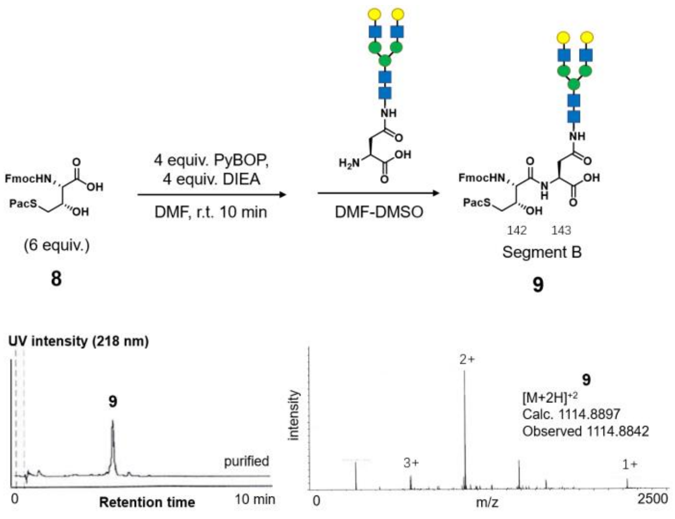

Figure S12. Synthesis of glycopeptide **9** with γ-mercapto-threonine.

γ-Mercapto-threonine **8** (5.6 mg, 0.014 mmol) was dissolved in dry DMF (228 μL). PyBOP (4.8 mg, 0.009 mmol) and DIEA (3.2 μL, 0.009 mmol) were added and stirred at room temperature for 10 minutes pre-activation. N-Asialo-glycosyl asparagine (2.0 mg, 2 μmol) was dissolved in DMF-DMSO = 7:3 solution (400 μL) and added into the reaction mixture. The mixture was stirred at room temperature for 1h and monitored by LC-MS. After completion of the reaction, chilled Et_2O was added for precipitation and the mixture was put on ice, then centrifuged (0 ^{o}C, 4000 rpm, 10 min) to collect precipitate. The precipitate was dissolved and purified by RP-HPLC (proteonavi C4 300Å 5 µm, 250 mm x 10 mm, Shiseido, 0.1% TFA: 0.1% TFA in 90% MeCN= 70/30 to 10/90 over 60 min at 2.5 mL/min) to afford γ-mercapto-L-threonyl-(N-asialo glycosyl) asparagine **9** in 50% yield after lyophilization. ESI-MS: calculated for $C_{93}H_{133}N_7O_{53}S$ 1114.8897 $[M+2H]^{+2}$, observed 1114.8842

Synthesis of γ-mercapto-L-threonyl-(N-asialo-glycosyl) asparagine S-trityl thioester 10

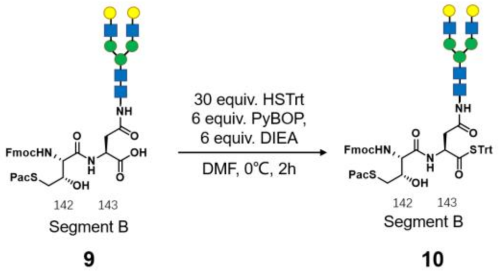

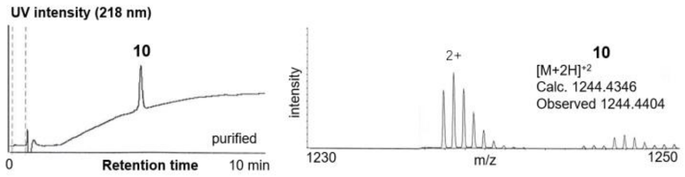

Figure S13. Synthesis of glycopeptide-thioester **10**.

To a solution of PyBOP (1.4 mg, 2.7 μmol) and TrtSH (3.7 mg, 13.5 μmol) in dry DMF (45 μL) was added compound **9** (1 mg, 0.45 μmol) on ice. The mixture was stirred at 0ºC for 10 min. DIEA (0.5 μL, 2.7 μmol) was added and the mixture was stirred at 0ºC until completion of the reaction monitored by LC-MS. Chilled Et_2O was added on ice for precipitation and the mixture was centrifuged (0ºC, 4000 rpm, 10 min) to collect precipitate. The precipitate was dissolved into 0.1% TFA and purified with RP-HPLC (proteonavi C4 300Å 5 μm, 250 mm x 10 mm, Shiseido, 0.1% TFA: 0.1% TFA in 90% MeCN= 60/40 to 10/90 over 60 min at 2.5 mL/min) to afford γ-mercapto-L-threonyl-(N-asialo-glycosyl) asparagine S-trityl thioester **10** in 45% yield after lyophilization. ESI-MS: calculated for $C_{112}H_{147}N_7O_{52}S_2$ 1244.4346 $[M+2H]^{+2}$, observed 1244.4404

Synthesis of γ-mercapto-L-threonyl-(N-asialo-glycosyl) asparagine thioacid 11

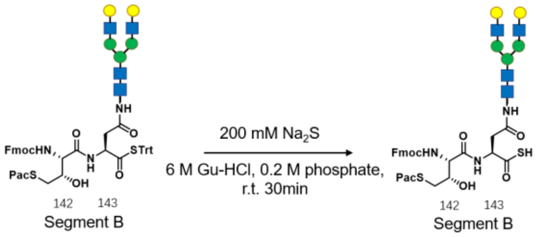

10

11

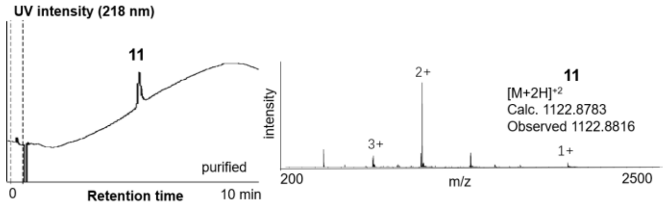

Figure S14. Synthesis of glycopeptide-SH **11**.

To a buffer solution (0.2 M phosphate, pH 6.8, 1 mL) containing Gn-HCl (6 M) and Na_2S (200 mM) was added compound **10** (1 mg, 0.45 μmol) and the mixture was shaken for 1 h. The mixture was directly purified with RP-HPLC (proteonavi C4 300Å 5 μm, 250 mm x 10 mm, Shiseido, 0.1% TFA: 0.1% TFA in 90% MeCN= 70/30 to 10/90 over 60 min at 2.5 mL/min) to afford γ-mercapto-L-threonyl-(N-asialo-glycosyl) asparagine thioacid **11** in 88%

yield after lyophilization. ESI-MS: calculated for $C_{93}H_{133}N_7O_{52}S_2$ 1122.8738 $[M+2H]^{+2}$, observed 1122.8816

E. coli expression of His6-SUMO-segment C (Cys144-Met183) 12

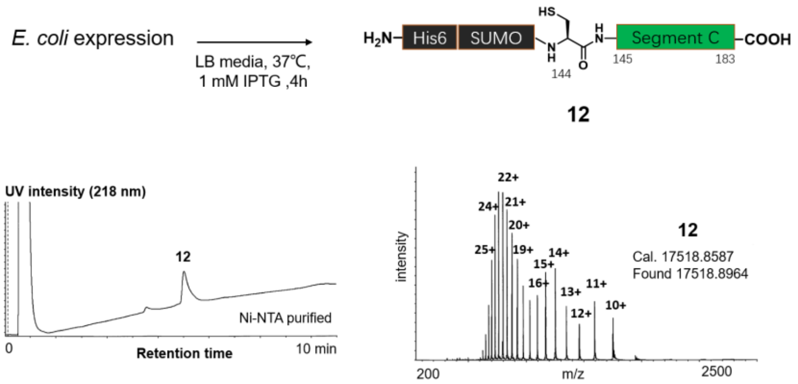

Figure S15. Preparation of His6-SUMO-segment C (Cys144-Met183) **12**.

The competent *Escherichia coli* strain BL21(DE3) (Nippon gene CO. LTD.) and pET302/NT-His vector (invitrogen^{TM}) encoded recombinant peptide were used for heat-shock transformation to afford recombinant *E. coli*., which was incubated on LB agar plate containing 100 µg/mL ampicillin (Unitech CO.) at 37 °C for 12 h. A single colony was picked up and inoculated into 10 ml LB media containing 100 µg/mL ampicillin and incubated at 37 °C for 12 h as the starter culture. The starter culture was inoculated at 1:100 dilution into 1 L LB media containing 100 µg/mL ampicillin and incubated at 37°C until OD600 reach 0.6-1.0 (3-4 h). IPTG was added in 1 mM final concentration to start overexpression and the 1 L media was further incubated at 37°C for 4 h. The 1 L expression media was centrifuged to collect *E. coli* pellet for cell lysis. After cell lysis, only inclusion body (precipitation) was collected by centrifugation. The inclusion body was treated with 6 M Gn-HCl buffer (pH 8.0) for denaturation. Denatured inclusion body was purified by Ni-NTA His_6 tag affinity purification then purified with RP-HPLC purification (proteonavi C4 300Å 5 µm, 250 mm x 10 mm, Shiseido, 0.1% TFA: 0.1% TFA in 90% MeCN= 70/30 to 10/90 over 60 min at 2.5 mL/min) to afford recombinant peptide **12** in 10 mg/L yield after lyophilization. ESI-MS: m/z calculated for $C_{763}H_{1221}N_{223}O_{234}S_8$: 17518.8587 $[M+1H]^{+1}$, observed 17518.8964 (deconvoluted). 649.7726 $[M+27H]^{+27}$, 674.7344 $[M+26H]^{+26}$, 701.6786 $[M+25H]^{+25}$, 730.8739 $[M+24H]^{+24}$, 762.6120 $[M+23H]^{+23}$, 797.2284 $[M+22H]^{+22}$, 835.1455 $[M+21H]^{+21}$, 876.8496 $[M+20H]^{+20}$, 922.9543 $[M+19H]^{+19}$, 974.1680 $[M+18H]^{+18}$, 1031.4308 $[M+17H]^{+17}$, 1095.8273 $[M+16H]^{+16}$, 1168.8190 $[M+15H]^{+15}$, 1252.2323 $[M+14H]^{+14}$, 1348.5074 $[M+13H]^{+13}$, 1460.7996 $[M+12H]^{+12}$, 1593.5178 $[M+11H]^{+11}$

SUMO cleavage: Synthesis of segment C (Cys144-Met183) 13

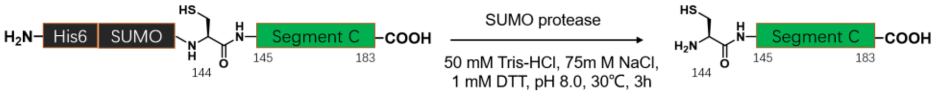

12 **13**

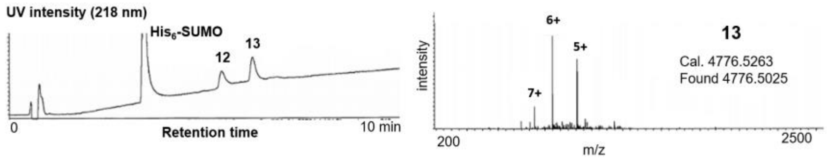

Figure S16. Preparation of segment C (Cys144-Met183) **13**.

To SUMO cleavage buffer (50 mM Tris-HCl, pH 8.0, 10 mL) containing NaCl (75 mM) and 1,4-Dithiothreitol (DTT, 1 mM) was dissolved His$_6$-SUMO-segment C (Cys144-Met183) **12** (10 mg, 0.57 μmol). A stock solution containing expressed SUMO protease was added. The reaction solution was shaken at 30 ^{o}C for 3 h until the completion of reaction monitored by LC-MS. The product precipitate as white solid during the reaction. The reaction solution was centrifuged (23°C, 4000 rpm, 10 min) to collect precipitate, which was dissolved in 6 M Gn-HCl then purified with RP-HPLC (proteonavi C4 300Å 5 µm, 250 mm x 10 mm, Shiseido, 0.1% TFA: 0.1% TFA in 90% MeCN= 60/40 to 10/90 over 60 min at 2.5 mL/min) to afford segment C (Cys144-Met183) **13** (1.5 mg, white solid) in 60% yield after lyophilization. ESI-MS: m/z calculated for $C_{211}H_{347}N_{61}O_{59}S$: 4776.5263 $[M+1H]^{+1}$, observed 4776.5025 (deconvoluted). 683.4540 $[M+7H]^{+7}$, 797.2000 $[M+6H]^{+6}$, 956.4450 $[M+5H]^{+5}$, 1195.3140 $[M+4H]^{+4}$

Synthesis of segment C [Cys144(S-Npys)-Met183] 14

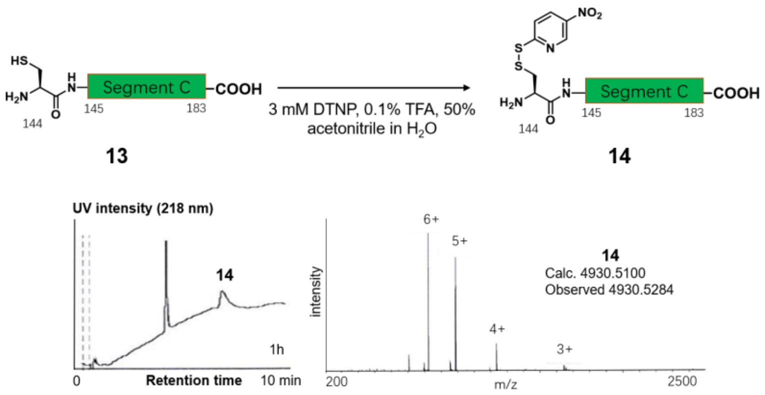

Figure S17. Preparation of segment C [Cys144(S-Npys)-Met183] **14**.

The segment C (Cys144-Met183) **13** (1.5 mg, 0.31 μmol) was dissolved in 0.1% TFA in acetonitrile-H_2O = 1:1 solution (8 mL). 2,2′-Dithiobis(5-nitropyridine) (DTNP) (1.9 mg, 6.2 μmol) was added and dissolved by sonication. The reaction mixture was shaken at room temperature for 1 h and monitored with LC-MS. After completion of the reaction, the mixture was purified with RP-HPLC (proteonavi C4 300Å 5 µm, 250 mm x 10 mm, Shiseido, 0.1% TFA: 0.1% TFA in 90% MeCN= 60/40 to 10/90 over 60 min at 2.5 mL/min) to afford segment C [Cys144(S-Npys)-Met183] **14** (1.5 mg, faint yellow solid) in 95% yield after lyophilization. ESI-MS: m/z calculated for $C_{216}H_{349}N_{63}O_{61}S_4$: 4930.5100 $[M+1H]^{+1}$, observed 4930.5284 (deconvoluted). 705.4632 $[M+7H]^{+7}$, 823.0431 $[M+6H]^{+6}$, 987.4552 $[M+5H]^{+5}$, 1233.8248 $[M+4H]^{+4}$

Thioacid capture ligation: Synthesis of segment BC [Thr142(S-Pac, *N*-Fmoc)-Met183, Asn143 *N*-glycosylated] 15

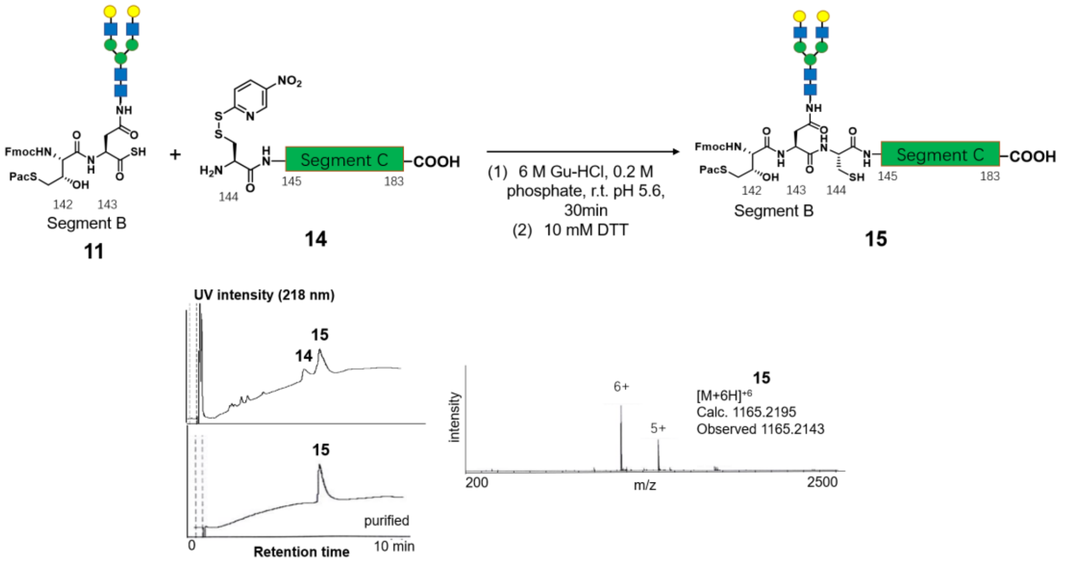

Figure S18 Synthesis of segment BC [Thr142(S-Pac, *N*-Fmoc)-Met183, Asn143 *N*-glycosylated] **15**.

Both compound **11** (3.5 mg, 1.56 μmol) and segment C [Cys144(S-Npys)-Met183] **14** (7.7 mg, 1.56 μmol) were dissolved in a buffer solution (0.2 M phosphate, pH 5.6, 1.56 mL) containing Gn-HCl (6 M) to start TCL reaction. The color of solution turned yellow which indicated the occurrence of reaction. The reaction was monitored by LC-MS. After completion, DTT aq. was added for quenching and the mixture was purified with RP-HPLC (proteonavi C4 300Å 5 μm, 250 mm x 10 mm, Shiseido, 0.1% TFA: 0.1% TFA in 90% MeCN= 60/40 to 10/90 over 60 min at 2.5 mL/min) to afford segment BC [Thr142(S-Pac, *N*-Fmoc)-Met183, Asn143 *N*-glycosylated] **15** (8.8 mg, white solid) in 80% yield after lyophilization. ESI-MS: m/z calculated for$C_{304}H_{478}N_{68}O_{111}S_4$: 1165.2195 $[M+6H]^{+6}$, observed 1165.2143, 999.4507 $[M+7H]^{+7}$, 1165.2143 $[M+6H]^{+6}$, 1398.8385 $[M+5H]^{+5}$, 1748.3033 $[M+4H]^{+4}$

Synthesis of segment BC [Thr142(SH, *N*-Fmoc)-Met183, Asn143 *N*-glycosylated] 16

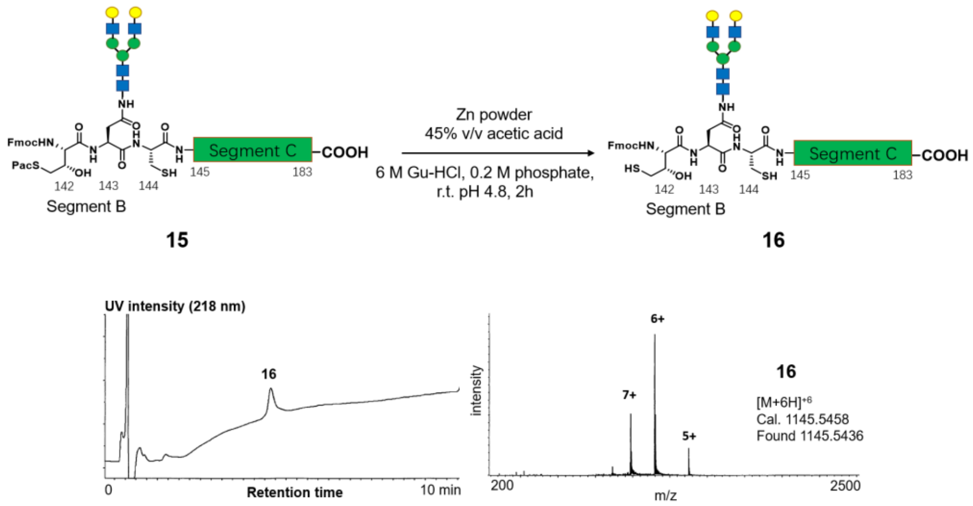

Figure S19. Synthesis of segment BC [Thr142(SH, *N*-Fmoc)-Met183, Asn143 *N*-glycosylated] **16**.

To a buffer solution (0.2 M phosphate, pH 4.8, 808 µL) containing Gn-HCl (6 M), acetic acid (660 µL, 45% v/v) and segment BC [Thr142(S-Pac, *N*-Fmoc)-Met183, Asn143 *N*-glycosylated] **15** (4.0 mg) was added pre-activated Zn powder (266 mg). The resultant solution was mixed by using vortex and sonication. The reaction was shaken at room temperature and monitored by LC-MS. After completion of the reaction, Zn powder was removed by centrifugation and filtration. The filtered solution was purified with RP-HPLC (proteonavi C4 300Å 5 µm, 250 mm x 10 mm, Shiseido, 0.1% TFA: 0.1% TFA in 90% MeCN= 60/40 to 10/90 over 60 min at 2.5 mL/min) to afford segment BC [Thr142(SH, *N*-Fmoc)-Met183, Asn143 *N*-glycosylated] **16** (3 mg, white solid) in 75% yield after lyophilization. ESI-MS: m/z calculated for $C_{296}H_{472}N_{68}O_{110}S_4$: 1145.5458 $[M+6H]^{+6}$, observed 1145.5436
982.5968 $[M+7H]^{+7}$, 1145.5436 $[M+6H]^{+6}$, 1375.2399 $[M+5H]^{+5}$, 1718.8042 $[M+4H]^{+4}$

Synthesis of segment BC [Thr142(SH)-Met183, Asn143 *N*-glycosylated] 17

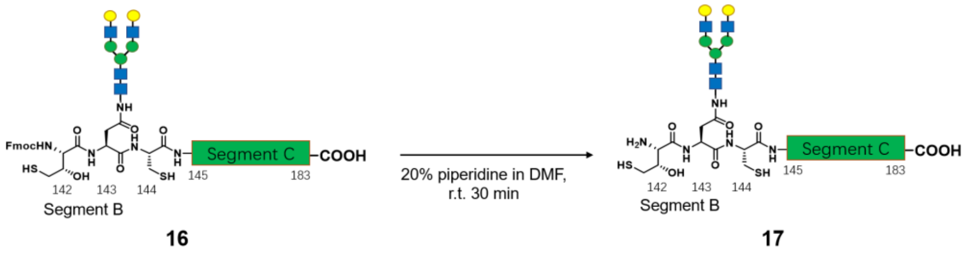

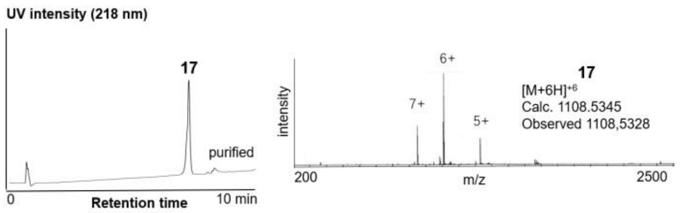

Figure S20. Synthesis of segment BC [Thr142(SH)-Met183, Asn143 *N*-glycosylated] **17.**

Segment BC [Thr142(SH, *N*-Fmoc)-Met183, Asn143 *N*-glycosylated] **16** (3 mg) was dissolved in 20% v/v piperidine-DMF (500 μL) and the reaction was shaken at room temperature for 20 min. After completion of the reaction as monitored with LC-MS, cold ether precipitation was carried out and the precipitate was collected by centrifugation (0℃, 4000rpm, 10min). RP-HPLC (proteonavi C4 300Å 5 μm, 250 mm x 10 mm, Shiseido, 0.1% TFA: 0.1% TFA in 90% MeCN= 60/40 to 10/90 over 60 min at 2.5 mL/min) afforded segment BC [Thr142(SH)-Met183, Asn143 *N*-glycosylated] **17** (2.3 mg, white solid) in 81% yield after lyophilization. ESI-MS: m/z calculated for $C_{281}H_{462}N_{68}O_{108}S_4$: 1108.5345 $[M+6H]^{+6}$, observed 1108.5328
950.7197 $[M+7H]^{+7}$, 1108.5328 $[M+6H]^{+6}$, 1330.6120 $[M+5H]^{+5}$, 1663.2682 $[M+4H]^{+4}$

Selenoester-mediated ligation: Synthesis of segment ABC [Val1-Met183, Asn143 *N*-glycosylated, Cys43(SPac), Cys49(SPac), Cys72(SPac), Cys82(SPac), Thr142(SH), Cys145] 18

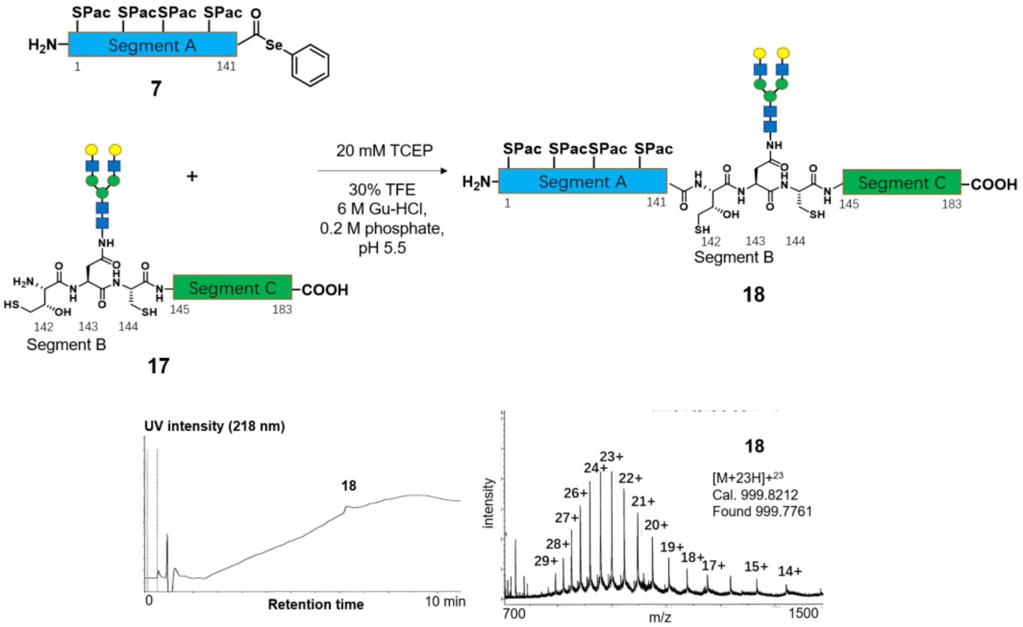

Figure S21. Synthesis of segment ABC [Val1-Met183, Asn143 *N*-glycosylated, Cys43(SPac), Cys49(SPac), Cys72(SPac), Cys82(SPac), Thr142(SH), Cys145] **18**.

To a buffer solution (0.2 M phosphate, pH 5.5, 25.2 μL) containing Gn-HCl (6 M), trifluoroethanol (TFE) (12.6 μL, 30% v/v) and TCEP (20 mM) was added 1 mM segment A [Val1-Thr141, Cys43(SPac), Cys49(SPac), Cys72(SPac), Cys82(SPac)]-SePh selenoester **7** (1 mg, 0.04 μmol) and Segment BC [Thr142(SH)-Met183, Asn143 *N*-glycosylated] **17** (0.56 mg, 0.08 μmol). The reaction was shaken at room temperature for around 12 h until completion of reaction monitored by LC-MS. A buffer solution (0.2 M

phosphate, pH 7.0, 60 μL) containing Gn-HCl (6 M) and TCEP (200 mM) was added and the reaction was directly purified with size-exclusive chromatography (TSKgel SuperSW3000 4.6 mm I.D. x 30 cm, 4 μm, TOSCH, 6 M guanidine-HCl, 0.1 M phosphate in ultrapure H2O, pH 6.5 over 35 min at 0.2 mL/min) to afford segment ABC [Val1-Met183, Asn143 *N*-glycosylated, Cys43(SPac), Cys49(SPac), Cys72(SPac), Cys82(SPac), Thr142(SH), Cys145] **18** in 10% yield (estimated by LC UV absorption) in 6 M Gu-HCl buffer. ESI-MS: m/z calculated for $C_{1003}H_{1601}N_{257}O_{335}S_{11}$ 999.8212 $[M+23H]^{+23}$, observed 999.7761

821.3813 $[M+28H]^{+28}$, 851.8168 $[M+27H]^{+27}$, 884.4938 $[M+26H]^{+26}$, 919.8432 $[M+25H]^{+25}$, 958.1339 $[M+24H]^{+24}$, 999.7761 $[M+23H]^{+23}$, 1045.1489 $[M+22H]^{+22}$, 1094.8955 $[M+21H]^{+21}$, 1149.5970 $[M+20H]^{+20}$, 1210.0350 $[M+19H]^{+19}$

Synthesis of NH-Trityl-(N-asialo glycosyl)-asparagine 19

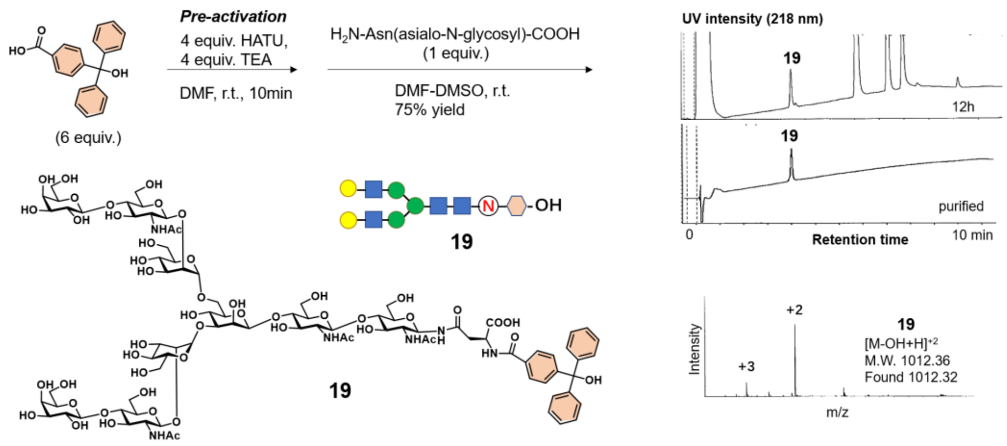

Figure S22. NH-Trityl-(N-asialo glycosyl)-asparagine **19**.

To a solution of 4-(diphenylhydroxymethyl) benzoic acid (4.5 mg, 15 μmol) and HATU (3.8 mg, 10 μmol) in dry DMF (150 μL) was added triethylamine (1.4 μL, 10 μmol). The mixture was stirred at room temperature for 10 min for pre-activation. The H_2N-Asn(N-asialo-glycan)-COOH (4.4 mg, 2.5 μmol) was dissolved in dry DMF:DMSO=7:3 solution (250 μL) and added into the reaction. The reaction mixture was stirred at room temperature and monitored by LC/MS until reaction finished. To the reaction mixture was added chilled diethyl ether for precipitation. After centrifugation, the precipitate was collected and purified by RP-HPLC (CAPCELL-PAK C18 300Å 5 μm, 250 mm x 10 mm, Shiseido, 0.1% TFA: 0.1% TFA in 90% MeCN= 90/10 to 10/90 over 60 min at 2.5 mL/min) to give compound **19** as white solid (3.5 mg, 75% yield). ESI-MS calculated for $C_{86}H_{124}N_6O_{50}$ 1012.3698 $[M-OH+H]^{+2}$, found 1012.3287

Synthesis of segment A [Val1-Thr141]-MesNa thioester 20

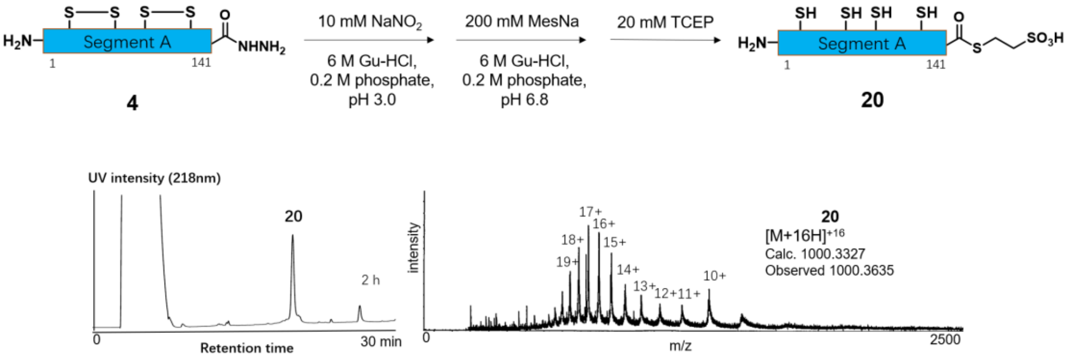

Figure S23. Synthesis of segment A [Val1-Thr141]-MesNa thioester **20.**

To a buffer solution (0.2 M phosphate, pH 3.0, 0.25 mL) containing Gu-HCl (6.0 M) and $NaNO_2$ (10 mM) was dissolved segment A (Val1-Thr141, Cys43-S-S-Cys49, Cys72-S-S-Cys82)-$NHNH_2$ **4** (2.0 mg, 0.5 mM). The reaction mixture was shaken at 0℃ for 20 min. A buffer solution (0.2 M phosphate, pH 7.0, 0.25 mL) containing Gu-HCl (6.0 M) and MesNa (200 mM) was added, then pH was adjusted to 6.8. The reaction was shaken at room temperature for 1-2 h and monitored with LC-MS. Finally, TCEP (20 mM) was added to the reaction mixture. The mixture was purified with RP-HPLC (proteonavi C4 300Å 5 µm, 250 mm x 4.6 mm, Shiseido, 0.1% TFA: 0.1% TFA in 90% MeCN= 70/30 to 20/80 over 30 min at 1.0 mL/min) to give segment A [Val1-Thr141]-MesNa thioester **20** (0.35 mg, white solid) in 45% yield for three steps after lyophilization. ESI-MS: m/z calculated for $C_{692}H_{1121}N_{189}O_{226}S_9$: 1000.3327 $[M+16H]^{+16}$, found 1000.3635
842.6804 $[M+19H]^{+19}$, 889.3950 $[M+18H]^{+18}$, 941.6593 $[M+17H]^{+17}$, 1000.3635 $[M+16H]^{+16}$, 1067.0435 $[M+15H]^{+15}$, 1143.1998 $[M+14H]^{+14}$, 1231.0637 $[M+13H]^{+13}$

Synthesis of segment A [Val1-Thr141, Cys43(STrtNglycan), Cys49(STrtNglycan), Cys72(STrtNglycan), Cys82(STrtNglycan)]-MesNa 21

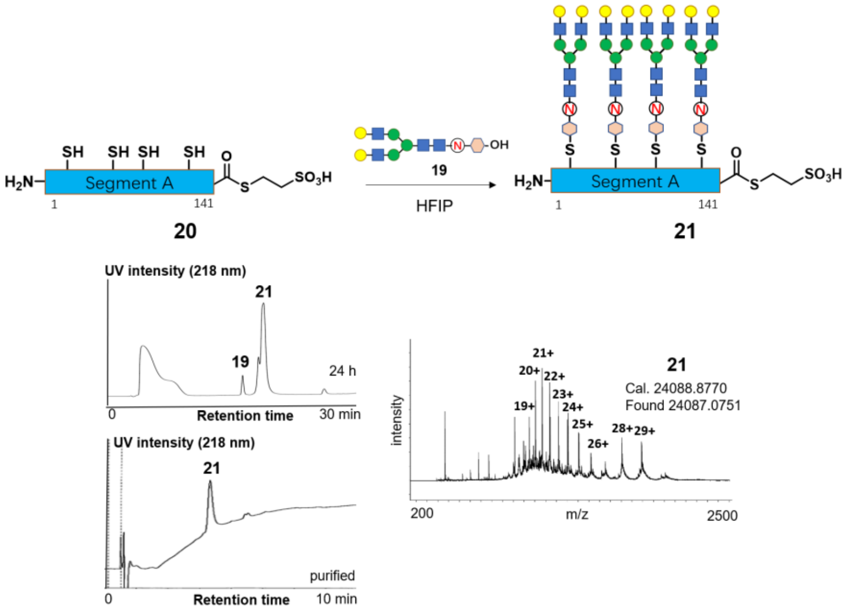

Figure S24. Synthesis of segment A [Val1-Thr141, Cys43(STrtNglycan), Cys49(STrtNglycan), Cys72(STrtNglycan), Cys82(STrtNglycan)]-MesNa **21**.

Compound **19** (1.0 mg, 0.49 μmol, 4 equiv./Cys) was dissolved in 1,1,1,3,3,3-hexafluoro-isopropanol (HFIP) (60 μL). To this fluorescent yellow solution was added peptide compound **20** (0.5 mg, 0.03 μmol). The reaction mixture was shaken at room temperature and monitored by LC/MS until reaction finished. To the reaction mixture was added chilled ethyl ether for precipitation. After centrifugation, the precipitate was collected and purified by RP-HPLC (proteonavi C4 300Å 5 μm, 250 mm x 4.6 mm, Shiseido, 0.1% TFA: 0.1% TFA in 90% MeCN= 70/30 to 10/90 over 60 min at 1.0 mL/min) to give segment A [Val1-Thr141, Cys43(STrtNglycan), Cys49(STrtNglycan), Cys72(STrtNglycan), Cys82(STrtNglycan)]-MesNa **21** as white solid (0.4 mg, 80% yield). ESI-MS: m/z calculated for $C_{1036}H_{1609}N_{213}O_{422}S_9$; 1417.9873 $[M+17H]^{+17}$, observed 1417.9802
1148.1483 $[M+21H]^{+21}$, 1205.4326 $[M+20H]^{+20}$, 1268.8642 $[M+19H]^{+19}$, 1339.2795 $[M+18H]^{+18}$, 1417.9802 $[M+17H]^{+17}$, 1506.5640 $[M+16H]^{+16}$, 1606.9436 $[M+15H]^{+15}$, 1721.6695 $[M+14H]^{+14}$, 1854.0554 $[M+13H]^{+13}$, 2008.2554 $[M+12H]^{+12}$

Native chemical ligation: Synthesis of segment ABC [Val1-Met183, Asn143 *N*-glycosylated, Cys43(STrtNglycan), Cys49(STrtNglycan), Cys72(STrtNglycan),

Cys82(STrtNglycan), Thr142(SH), Cys145] 22

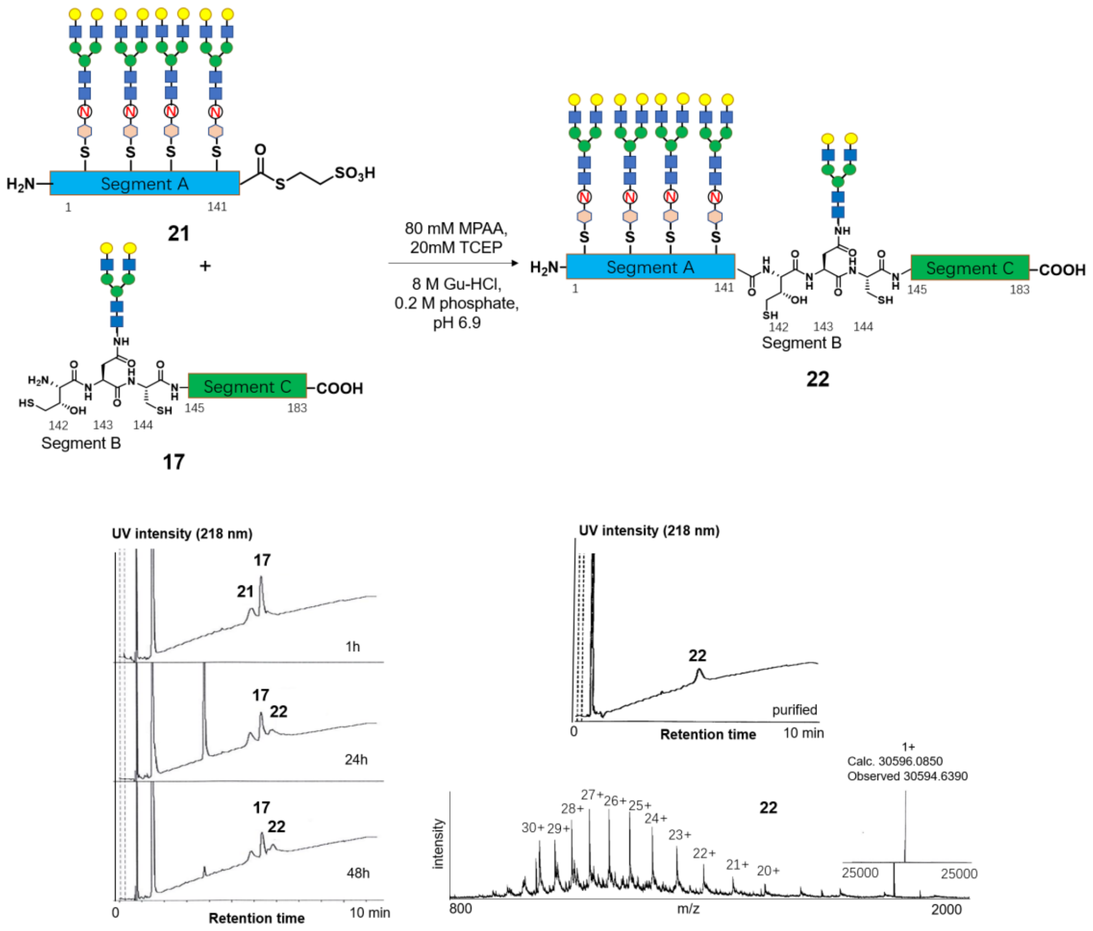

Figure S25. Synthesis of segment ABC [Val1-Met183, Asn143 *N*-glycosylated, Cys43(STrtNglycan), Cys49(STrtNglycan), Cys72(STrtNglycan), Cys82(STrtNglycan), Thr142(SH), Cys145] **22.**

To a buffer solution (0.2 M phosphate, pH 6.9, 21 μL) containing Gu-HCl (8 M), MPAA (80 mM) and TCEP (20 mM) was added segment A [Val1-Thr141, Cys43(STrtNglycan), Cys49(STrtNglycan), Cys72(STrtNglycan), Cys82(STrtNglycan)]-MesNa **21** (1 mg, 0.04 μmol, 2 mM) and Segment BC [Thr142(SH)-Met183, Asn143 *N*-glycosylated] **17** (0.56 mg, 0.08 μmol, 4 mM). The reaction was shaken at room temperature for around 48 h until completion of reaction monitored by LC-MS. A buffer solution (0.2 M phosphate, pH 6.9, 2 μL) containing Gn-HCl (6 M) and TCEP (200 mM) was added every 12 h. The reaction was directly purified with size-exclusive chromatography (TSKgel SuperSW3000 4.6 mm I.D. x 30 cm, 4 μm, TOSCH, 6 M guanidine-HCl, 0.1 M phosphate in ultrapure H2O, pH 6.5 over 35 min at 0.2 mL/min) to afford segment ABC [Val1-Met183, Asn143 *N*-glycosylated, Cys43(STrtNglycan), Cys49(STrtNglycan), Cys72(STrtNglycan), Cys82(STrtNglycan), Thr142(SH), Cys145] **22** (50% yield, estimated by LC UV absorption: half consumption of substrate **21**:0.04 μmol) in 6 M Gu-HCl buffer. This product was used for the next step without further purification. ESI-MS: m/z calculated for $C_{1315}H_{2065}N_{281}O_{527}S_{11}$: 1275.8231 $[M+24H]^{+24}$, observed 1275.8273

1020.8228 [M+30H]$^{+30}$, 1056.0225 [M+29H]$^{+29}$, 1093.6473 [M+28H]$^{+28}$, 1134.1366 [M+27H]$^{+27}$, 1177.6999 [M+26H]$^{+26}$, 1224.7964 [M+25H]$^{+25}$, 1275.8273 [M+24H]$^{+24}$, 1331.2507 [M+23H]$^{+23}$, 1391.6294 [M+22H]$^{+22}$

Desulfurization: Synthesis of segment ABC [Val1-Met183, Asn143 *N*-glycosylated, Cys43(STrtNglycan), Cys49(STrtNglycan), Cys72(STrtNglycan), Cys82(STrtNglycan)] 23

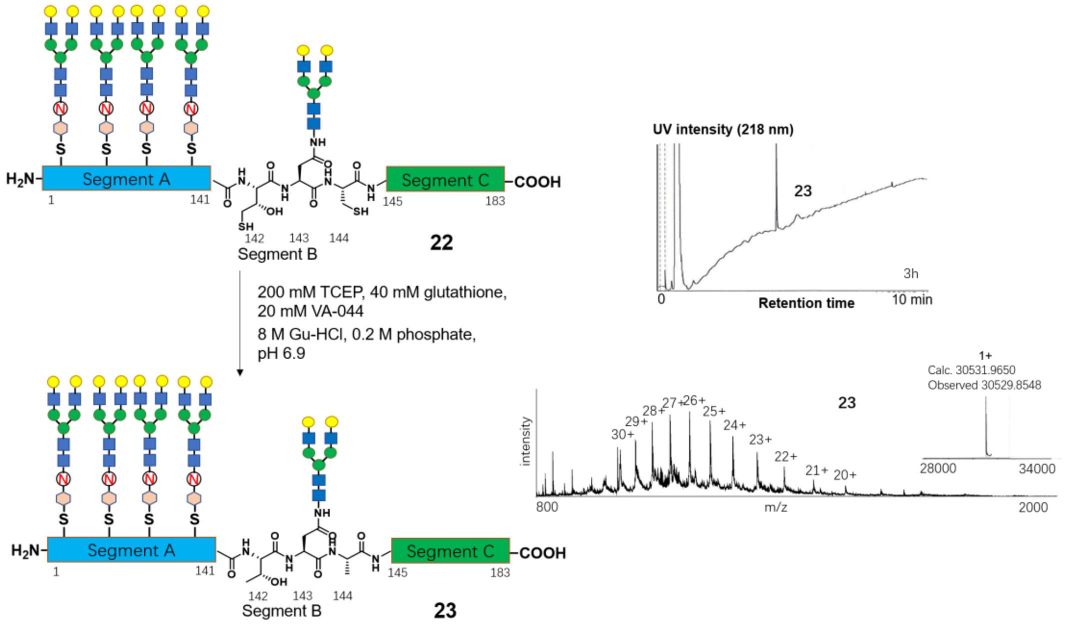

Figure S26. Synthesis of segment ABC [Val1-Met183, Asn143 *N*-glycosylated, Cys43(STrtNglycan), Cys49(STrtNglycan), Cys72(STrtNglycan), Cys82(STrtNglycan)] **23**.

To a buffer solution (0.2 M phosphate, pH 6.9, 300 µL) containing Gu-HCl (6.0 M), glutathione (3.7 mg, 40 mM), TCEP (17.2 mg, 200mM) was added segment ABC (Val1-Met183, Asn143 *N*-glycosylated, Thr142(SH), Cys145) **22** in solution after size-exclusive chromatography. VA-044 (1.9 mg, 20 mM) was added and the reaction mixture was shaken at room temperature until completion of reaction monitored by LC-MS. The reaction mixture was purified by RP-HPLC (proteonavi C4 300Å 5 µm, 250 mm x 4.6 mm, Shiseido, 0.1% TFA: 0.1% TFA in 90% MeCN= 70/30 to 10/90 over 60 min at 1.0 mL/min) to afford segment ABC [Val1-Met183, Asn143 *N*-glycosylated, Cys43(STrtNglycan), Cys49(STrtNglycan), Cys72(STrtNglycan), Cys82(STrtNglycan)] **23** in 78% yield after lyophilization (ca 0.47 mg). m/z calculated for $C_{1315}H_{2065}N_{281}O_{527}S_9$: 1175.2628 [M+26H]$^{+26}$, observed 1175.2619 1053.7579 [M+29H]$^{+29}$, 1091.3467 [M+28H]$^{+28}$, 1131.7854 [M+27H]$^{+27}$, 1175.2619 [M+26H]$^{+26}$, 1222.2298 [M+25H]$^{+25}$, 1273.0551 [M+24H]$^{+24}$, 1328.3897 [M+23H]$^{+23}$, 1388.7599 [M+22H]$^{+22}$

Synthesis of Segment ABC (Val1-Met183, Asn143 *N*-glycosylated) 24 and recovering glycan-tag 19.

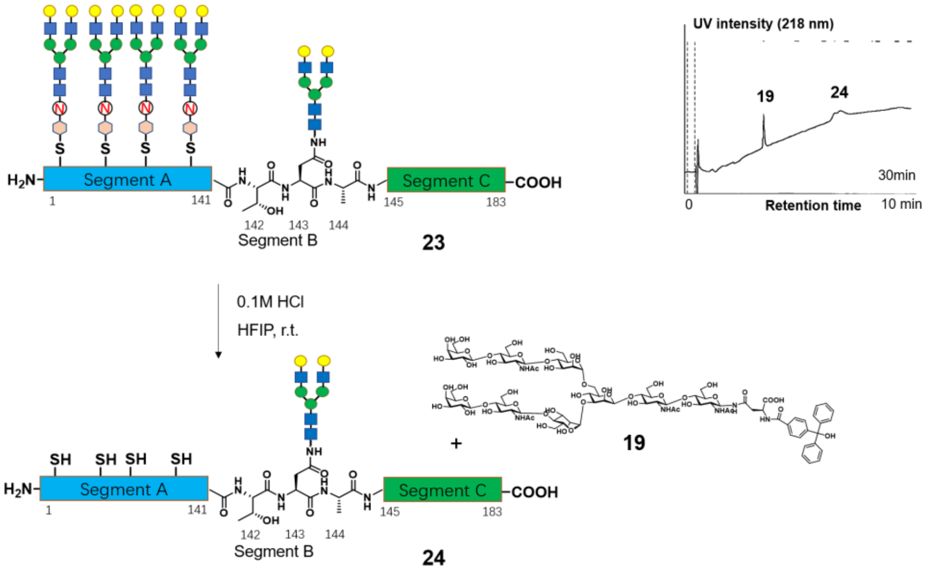

Figure S27. Segment ABC (Val1-Met183, Asn143 *N*-glycosylated) **24** and recovering glycan-tag **19**.

Segment ABC [Val1-Met183, Asn143 *N*-glycosylated, Cys43(STrtNglycan), Cys49(STrtNglycan), Cys72(STrtNglycan), Cys82(STrtNglycan)] **23** (1.0 mg, 0.03 µmol) was dissolved in HFIP (50 µL) containing HCl (0.1 M). The reaction mixture was shaken at room temperature for 30 min and monitored with LC-MS to give Segment ABC (Val1-Met183, Asn143 *N*-glycosylated) **24**. HPLC profile shows two peaks about compound **24**. This indicated partial folding immediately started and mass spectroscopy supported disulfide bond formation. The solution was directly used for the next folding reaction. *The glycan tag **19** could be recovered in good yield in this process and was found to be used for the next glycan tag-installation.*

Synthesis of folded 143glycosyl-IL-6 by *in vitro* folding

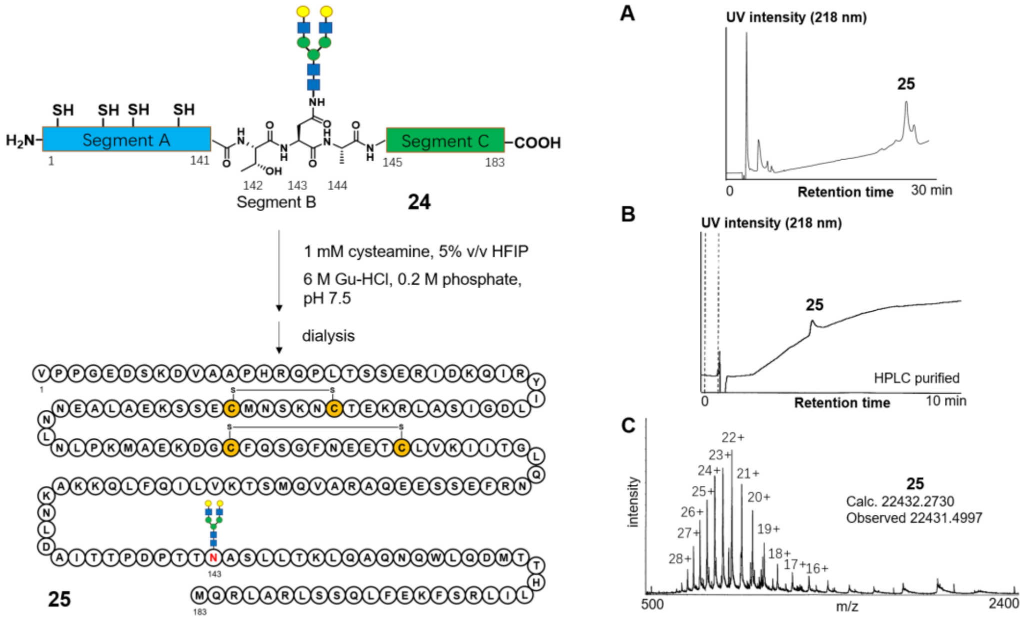

Figure S28. Folding of 143glycosyl-IL-6 **25**. A: crude HPLC of **25**; B: HPLC profile of the purified **25**; C: mass spectroscopy of purified **25**.

To a solution of HFIP (50 μL) containing Segment ABC (Val1-Met183, Asn143 *N*-glycosylated) **24** was added buffer solution (50 mM Tris-HCl, pH 8.0, 950 μL) containing Gu-HCl (6 M) and cysteamine (1 mM). The solution was vortexed and shaken at room temperature for 24 h in order to include air and monitored with LC-MS. The solution was dialyzed through Tube-O-DIALYZER^{TM} Mixed, 8kDa MWCO, G-BIOSCIENCES into buffer solution (50 mM Tris-HCl, pH 7.5) for 2 h and purified by RP-HPLC (proteonavi C4 300Å 5 µm, 250 mm x 4.6 mm, Shiseido, 0.1% TFA: 0.1% TFA in 90% MeCN= 70/30 to 10/90 over 60 min at 1.0 mL/min) to afford IL-6 (Asn143 *N*-glycosylated) **25** (0.25 mg, white solid) in 33% yield. m/z calculated for $C_{971}H_{1573}N_{257}O_{331}S_{9}$: 1020.6409 $[M+20H]^{+20}$, observed 1020.6446

831.8126 $[M+27H]^{+27}$, 863.7714 $[M+26H]^{+26}$, 898.2768 $[M+25H]^{+25}$, 935.6922 $[M+24H]^{+24}$, 976.3853 $[M+23H]^{+23}$, 1020.6446 $[M+22H]^{+22}$, 1069.2507 $[M+21H]^{+21}$, 1122.6042 $[M+20H]^{+20}$, 1181.6444 $[M+19H]^{+19}$, 1247.2254 $[M+18H]^{+18}$, 1320.6328 $[M+17H]^{+17}$

Disulfide bonds mapping

The disulfide mapping was carried out by using Lysyl Endopeptidase (Mass spectrometry grade, wako). The folded IL-6 in 50% acetonitrile solution (0.5 μL) containing 0.1% TFA was diluted in a buffer solution (2.0 mM Tris-HCl buffer, pH 8.0, 16 μL). A buffer solution (4.0 μL) containing Lysyl endopeptidase was added, and the mixture was incubated at room temperature for 20 min and analyzed by LC/MS.

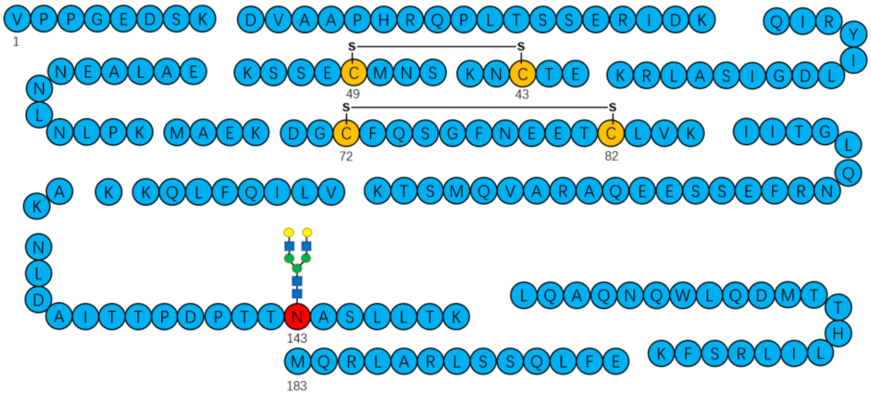

Possible fragments digested by Lysyl Endopeptidase

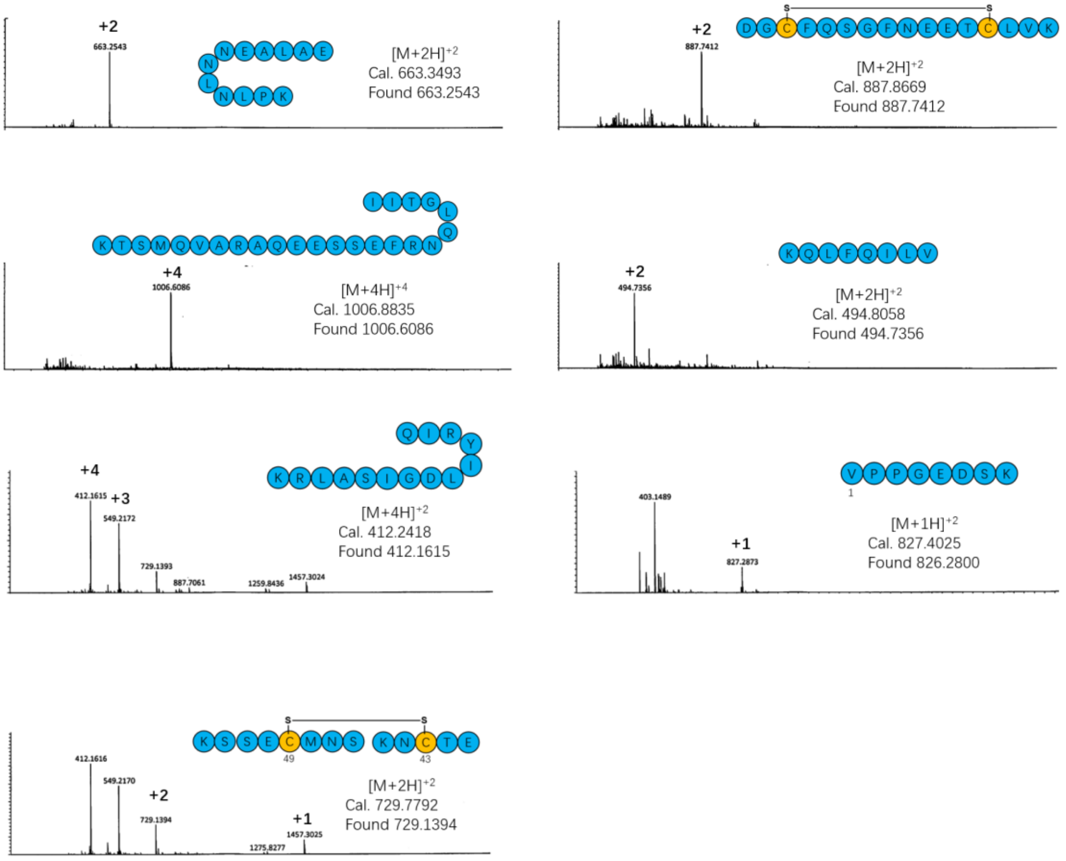

Observed mass of fragments

Sequence	calculated	found
VPPGEDSK (1-8)	827.4025 $[M+1H]^{+2}$	826.2800
QIRYILDGISALRK (27-40)	412.2418 $[M+4H]^{+2}$	412.1615
ET**C**NKSNM**C**ESSK (41-53)	729.7792 $[M+2H]^{+2}$	729.1394
EALAENNLNLPK (54-65)	663.3493 $[M+2H]^{+2}$	663.2543
DG**C**FQSGFNEET**C**LVK (70-85)	887.8669 $[M+2H]^{+2}$	887.7412
IITGLLEFEVYLEYLQNRFESSEEQARAVQMSTK (86-109)	1006.8835 $[M+4H]^{+4}$	1006.6086
VLIQFLQK (110-117)	494.8058 $[M+2H]^{+2}$	494.7356

Figure S29. Disulfide bonds mapping of 143glycosyl-IL6 **25.**

Synthesis and installation of lactose-based hydrophilic tag

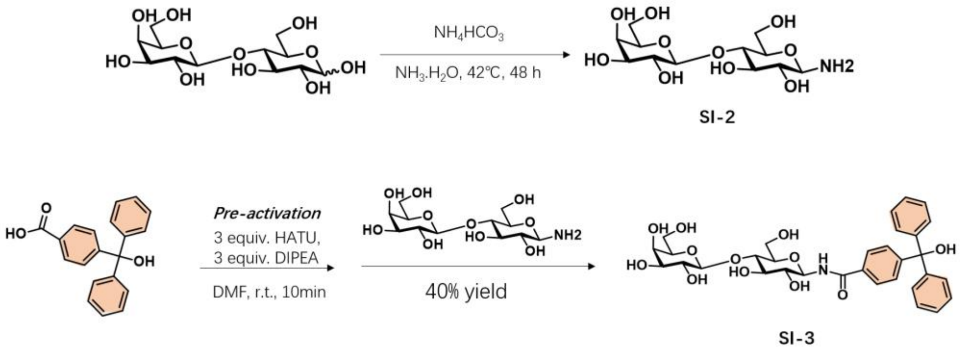

Figure 30. Synthesis and installation of lactose-based hydrophilic tag.

Compound **SI-2** was synthesized from lactose **SI-1** according to a reported protocol [Fenger T. H. et al. *ChemBioChem* **2015**, 16, 575 – 583]. Compound **SI-3** was synthesized via coupling reaction using HATU/DIPEA.

The installation of lactose-based hydrophilic tag **SI-3** was examined on a model peptide containing two cysteines as modification sites. The reaction was carried out in HFIP to afford target product. The LC result indicated that the hydrophobicity of model peptide was remarkably increased due to lactose-based tag, according to the changing of retention time.

Installation of Lys6 hydrophilic tag

Trityl-polyLys$_6$ (1.0 mg, 0.49 µmol, 16 equiv., 4 equiv./Cys) was dissolved in 1,1,1,3,3,3-hexafluoro-isopropanol (HFIP) (120 µL). To this fluorescent yellow solution was added peptide compound **20** (0.5 mg, 0.03 µmol, 1 equiv.). The reaction mixture was shaken at room temperature and monitored by LC/MS for 96 h. To the reaction mixture was added chilled ethyl ether for precipitation. After centrifugation, the precipitate was collected and purified by RP-HPLC (proteonavi C4 300Å 5 µm, 250 mm x 4.6 mm, Shiseido, 0.1% TFA: 0.1% TFA in 90% MeCN= 70/30 to 10/90 over 60 min at 1.0 mL/min) to give compound **SI-4** as main product (Lys6 tag-installation positions were not determined). Segment A bearing three 4/3/2 polyLys$_6$ tags were isolated in 1:4:5 ratio.

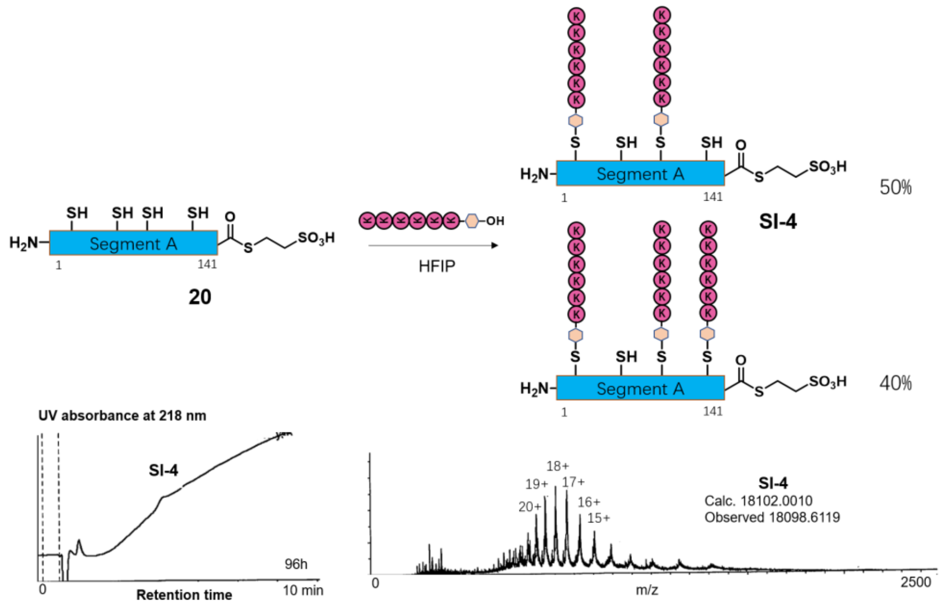

Figure S31. Installation of Lys-tag to segment A.

We hypothesized that such incomplete installation was caused by repelling effect due to electrostatic interaction between cationic Lys side chains. When modification sites are close (5-9 AA), the reaction efficiency is decreased a lot. *Lys6 tag-installation positions were not determined*.

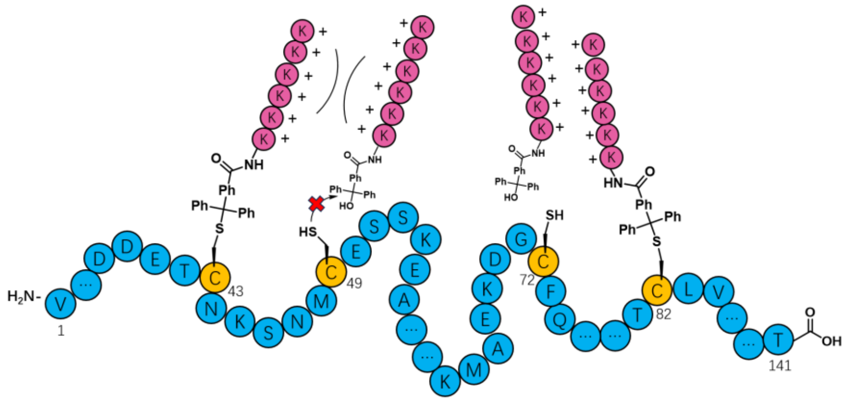

Figure S32. Proposed mechanism for low installation yield of polyLys$_6$ tag.

ANS fluorescence assay

The substrate was dissolved in a buffer solution (50 mM Tris-HCl, pH 7.5) to 10 µM concentration and ANS (8-Anilino-1-naphthalenesulfonic acid, TCI) was added (50 µM, 5 equiv.). The sample was incubated at room temperature for 30 min. ANS fluorescence assay was measured on JASCO fp-6500 spectrofluorometer at emission measurement mode. Parameter: 0.5 seconds response, low sensitivity, excitation wavelength 369 nm, emission wavelength 400-600 nm, resolution 1 nm, scan speed 200 nm/min. Y axis indicate the intensity of ANS fluorescence.

Compound **2**

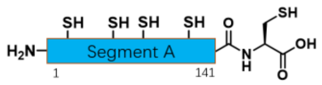

2

Compound **3**

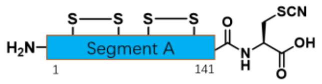

3

Compound **21**

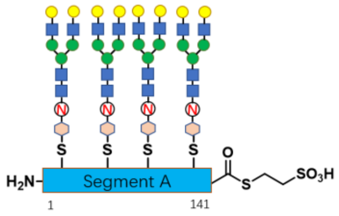

21

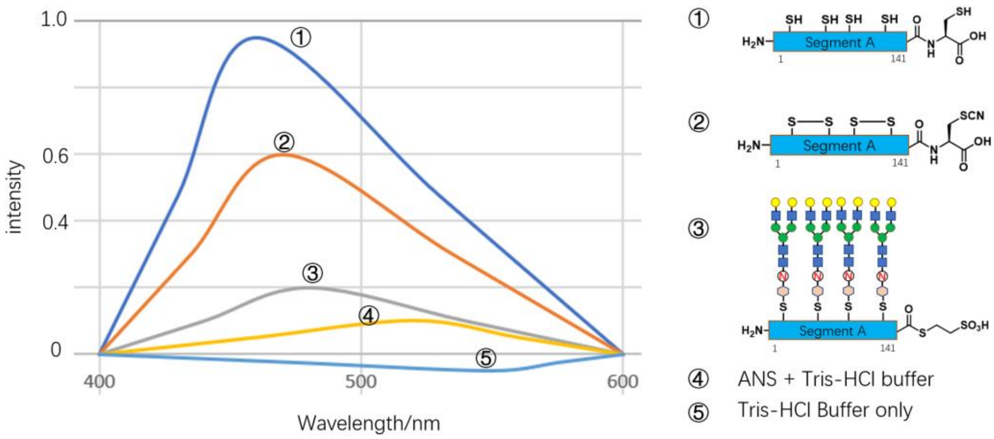

Figure S33. Evaluation of hydrophobicity of peptide segments with 8-Anilino-1-naphthalenesulfonic acid.

TF1 cell proliferation assay

The activity of IL-6 was determined using the TF1, a human erythroblast cell line, proliferation assay as described (Eur. J. Immunology, https://onlinelibrary.wiley.com/doi/epdf/10.1002/eji.1830240143). Briefly, eight thousand TF1 cells were plated in a 96-well plate with the indicated concentration of IL6 and incubated for 72 hours. Cell proliferation was measured using CellTiter-Glo 2.0 (Promega Japan, Tokyo, Japan), which quantifies the amount of cellular ATP produced by metabolically active cells. Commercially available recombinant hIL-6 without N-glycosylation was used as control (R and D Systems Japan : #206-IL, Tokyo, Japan, Recombinant Human IL-6, bio-techne, reconstitute at 100-200 µg/mL in sterile PBS containing at least 0.1% human or bovine serum albumin). Synthetic 143glycosyl-IL-6 was diluted and prepared to final concentration of 10000.0 pg/100 µL, 2500.0 pg/100 µL, 625.0 pg/100 µL, 156.3 pg/100 µL, 39.1 pg/100 µL, 9.77 pg/100 µL, 2.44 pg/100 µL, 0.61 pg/100 µL, 0.15 pg/100 µL. Recombinant hIL-6 was diluted and prepared to final concentration of 1000.0 pg/100 µL, 250.0 pg/100 µL, 62.5 pg/100 µL, 15.6 pg/100 µL, 3.91 pg/100 µL, 0.98 pg/100 µL, 0.24 pg/100 µL, 0.06 pg/100 µL, 0.015 pg/100 µL.

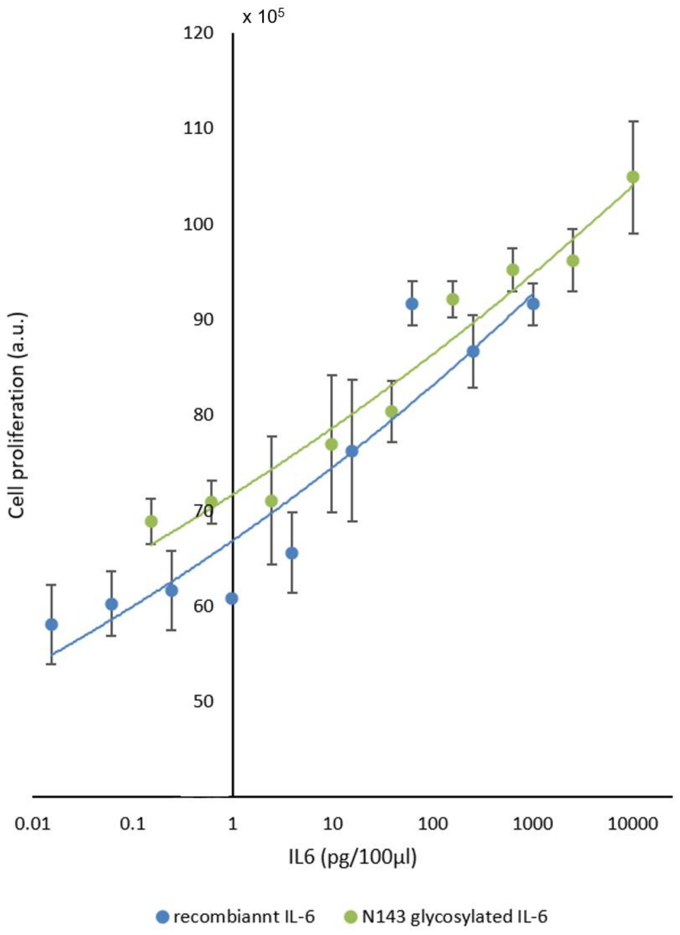

Figure S34. Cell proliferation assays with synthetic 143glycosyl-IL6 **25**.

MD Simulation

In order to make an initial structure, the coordination of PDB (ID: 1il6) was used. A helix area from 141 to 154 positions was rotated approximately 180° in Chimera soft wear (https://www.cgl.ucsf.edu/chimera/). Then structure of only the loop part was firstly minimized by the Chimera, because there was a gap due to the rotation around 141-154 residues. Subsequently other polypeptide backbone parts were constrained and minimized. Glycans were added to this structure by Glycam website (https://glycam.org) and was minimized through this web system to make the initial structure for MD calculations.

MD calculations were performed using GROMACS for 100 nsec with NPT Ensemble (1atom, 303.15K) and Force Field CHARMM36m. GROMACS input files were created using the Solution Builder in the Input Generator of CHARMM-GUI. The structure of 143glycosyl-IL6 was abstracted at every 10 ns and the resultant 10 structure were used for superimposed structure as shown in Figure 10.

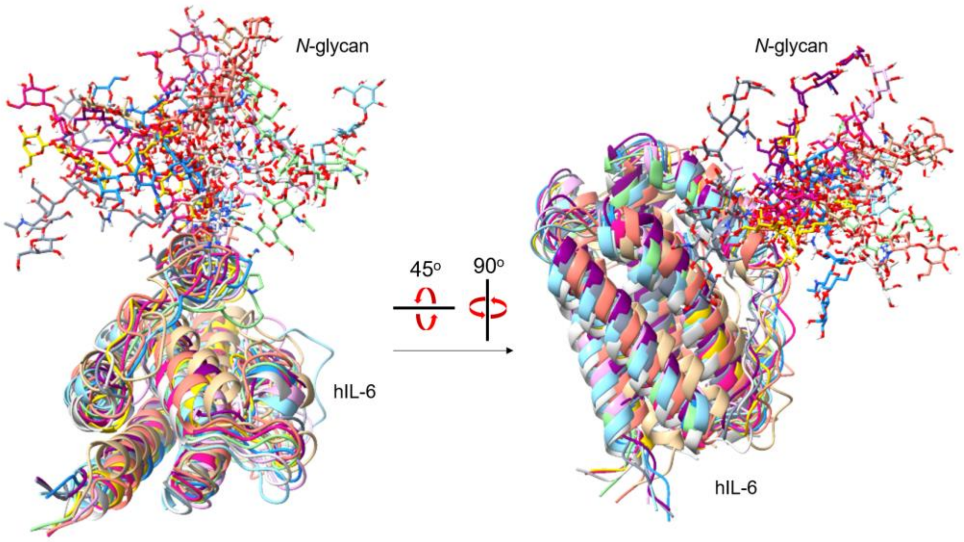

Figure S35. Superimposed structure of ten 143glycosyl-IL6.

NMR spectra of compound 8

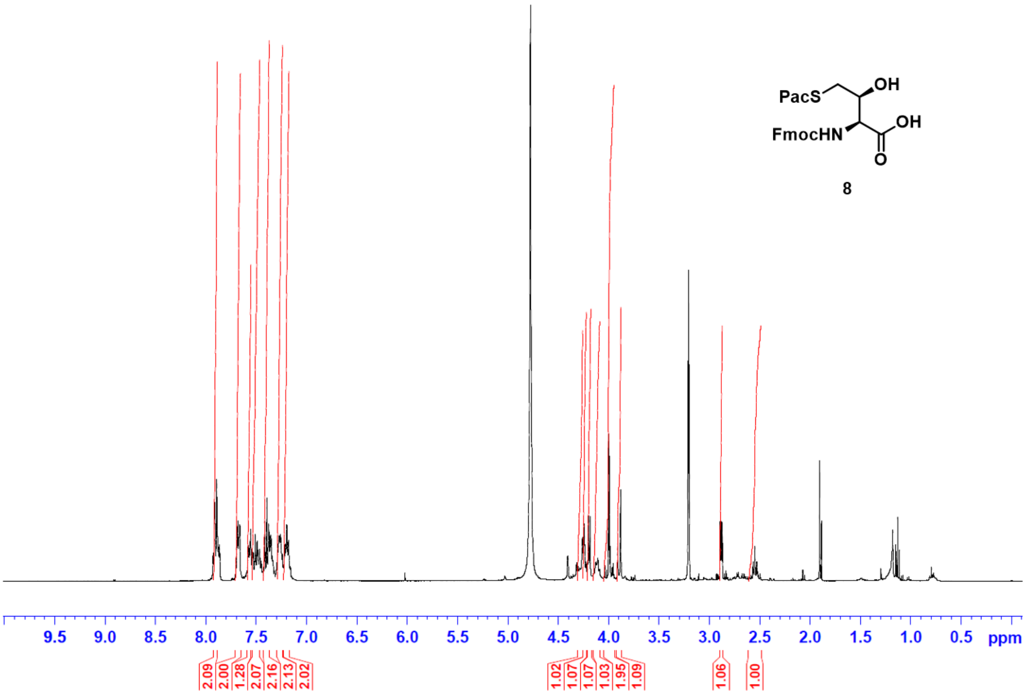

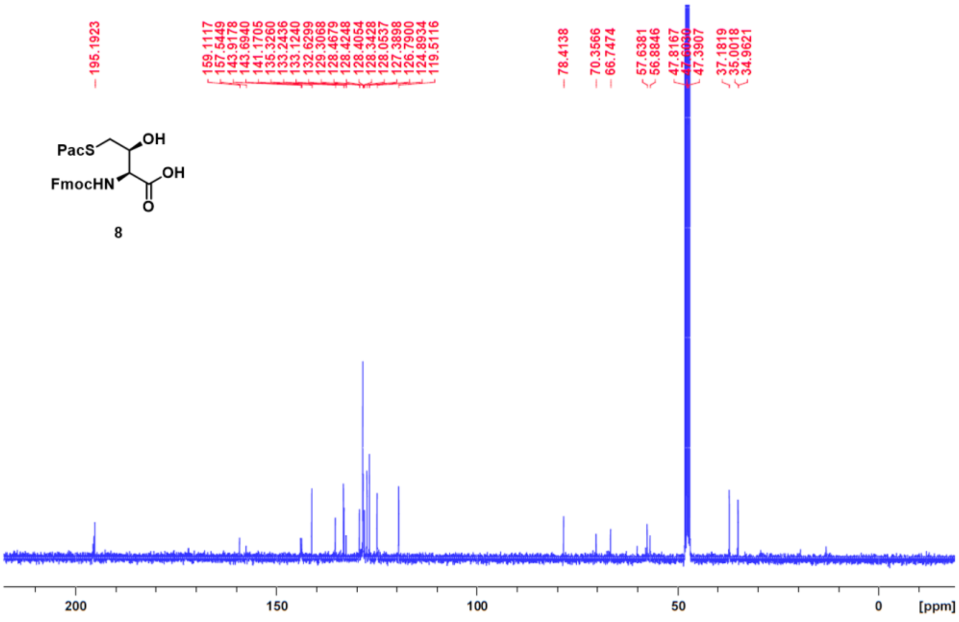

Figure S36. ^{1}H NMR and ^{13}C NMR spectra of compound mercapto threonine **8**.

Plasmids information.

We ordered plasmid which already included His tag sequence, to Thermo Fischer Scientific. Toward the plasmid, we ordered to insert the sequence with PmlI/SUMO-tag sequence/target sequence/BamHI where PmlI and BamHI are the sequences of restriction enzymes. We used the following SUMO-Tag sequence. If the unsuitable sequences are included in the target sequence, we changed the restriction enzyme site in the commercially available Plasmid.

SUMO tag sequence:
MSDSEVNQEAKPEVKPEVKPETHINLKVSDGSSEIFFKIKKTTPLRRLMEAFAKRQGKEMDSLRFLYDGIRIQADQTPEDLDMEDNDIIEAHREQIGG.
In addition, the polyhistidine tag (His_6) was engineered to the N-terminus of the SUMO sequence.

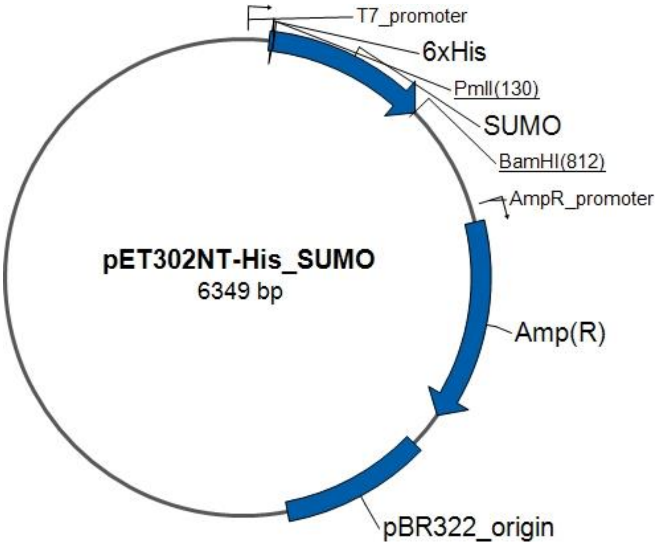

Figure S37. Plasmid construct purchased from ThermoFischer Scientific

Insertion of the target sequence into the plasmid.

The target sequence of N-terminal (1-141-Cys) and C-terminal (144-183) along with SUMO-tag were individually inserted by use of restriction enzymes. In terms of SUMO-protease, we prepared the plasmid and expressed in E.coli. After His-tag purification we used it for the cleavage of SUMO-tag from the resultant peptides and then used the N-terminal (1-141-Cys) and C-terminal (144-183) peptides for IL-6 synthesis.

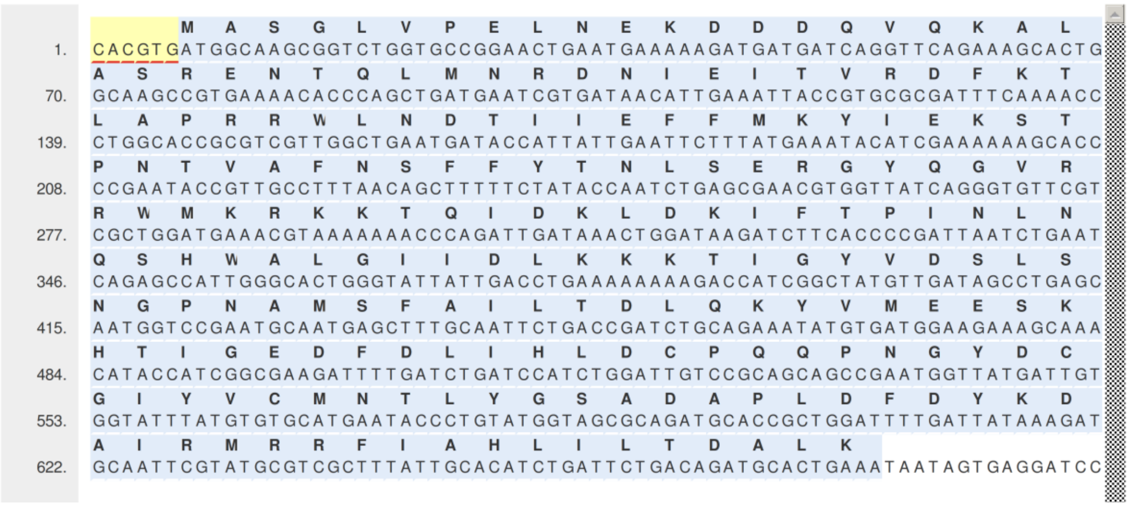

Figure S38. Sequence of SUMO protease.

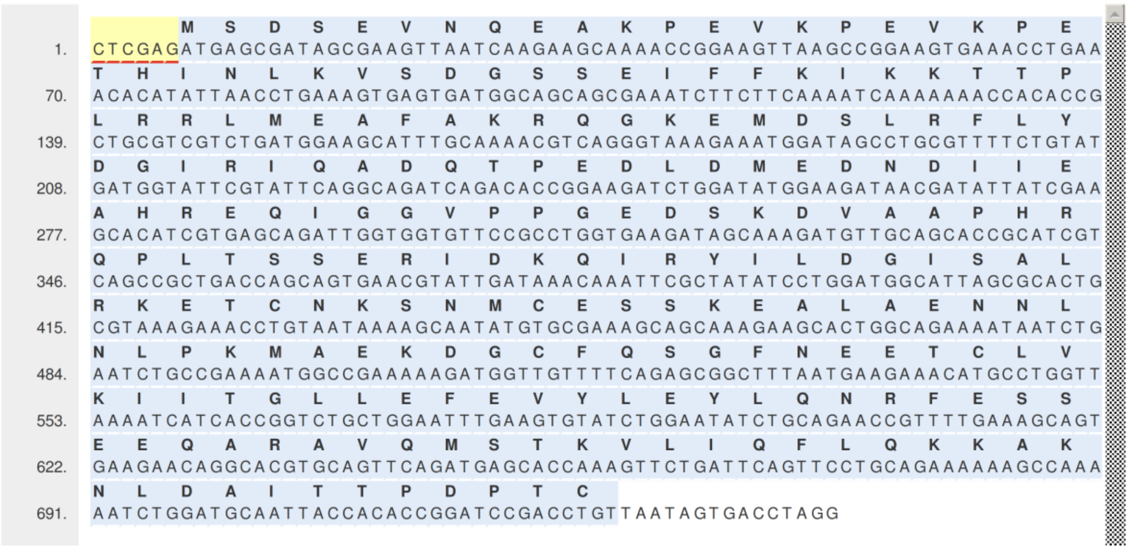

Figure S39. Sequence of fusion protein containing segment A (1-141-cys) which is SUMO-Tag and C-terminal peptide sequence.

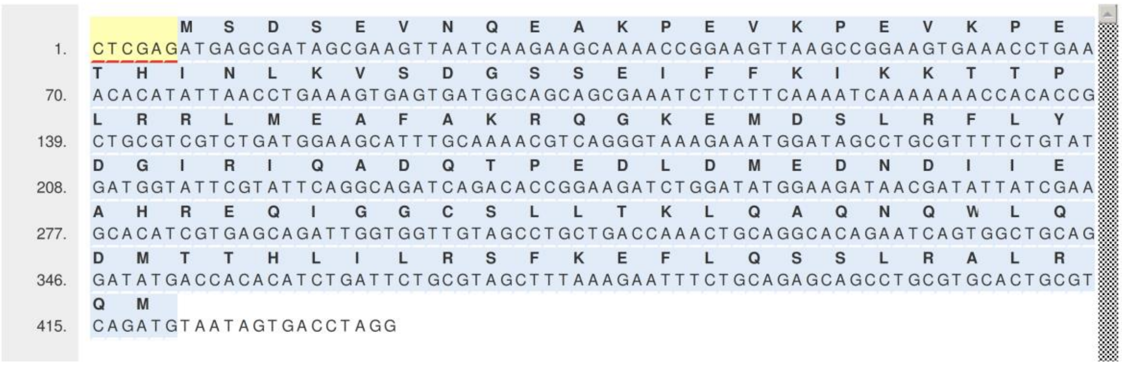

Figure S40. Sequence of fusion protein containing segment C (144-183) which is SUMO-Tag and C-terminal peptide sequence.

Typical protocol for transformation of *E. coli* and recombinant expression of fusion peptide

The plasmid containing desired genes in the pET vector was transformed into *E. coli* BL21(DE3) strain by heat-shock treatment. The plasmid (0.5 μL, 0.5 μg/μL) was added to a solution including *E. coli* cells (10 μL), treated at 42 ^{o}C for 45 sec, and immediately transferred to an ice bath and left for 5 min. Competent cells were obtained by plating the transformed cells on an LB agar plate containing ampicillin at 37 ^{o}C overnight. One of the colonies from the plate was used to make glycerol stock that can be used for the preparation of starter culture for protein expression. A starter culture was obtained by inoculating 5 mL of LB broth containing ampicillin (0.1 g/mL) with 0.1 mL of glycerol stock at 37 ^{o}C overnight. The starter culture was then used to inoculate LB media (1 L) containing ampicillin (0.1 g/mL) and the media was incubated at 37 ^{o}C until the optical density (OD) at 600 nm reached 0.75 OD as an intact direct solution. The culture media was then cooled to room temperature and induced with IPTG at the final concentration of 0.1 g/mL was added and incubated at 37 ^{o}C for 4 h. *E. coli* cells were collected by centrifugation at 8000 rpm for 10 min at 4 ^{o}C. The precipitated cells were gently dissolved in an appropriate amount of lysis buffer on an ice bath. The composition of the lysis buffer is 50 mM of sodium phosphate, pH 7.8, 400 mM NaCl, 100 mM KCl, 10% glycerol, 0.1% triton X-100, 10 mM imidazole, referring to the protocol provided by Invitrogen. The cells were lysed by ultrasonication for 1 min with pauses on an ice bath four times. This was subjected to centrifugation at 10000 rpm for 10 min at 4 ^{o}C. The fusion peptide, a soluble peptide, remains in the buffer, so the precipitated cell debris was discarded. Purification of fusion peptide was performed using cOmplete His-tag resin under native conditions using varying compositions of Buffer A (50 mM NaH_2PO_4, pH 8.0, 300 mM NaCl) and Buffer B (50 mM NaH_2PO_4, pH 8.0, 300 mM NaCl, 250 mM imidazole), referring to the protocol provided by Roche. The eluent containing fusion peptide was purified using reverse-phase HPLC (Proteonavi C4 Φ10 × 250 mm, 0.1% TFA : 0.1% TFA in 90% MeCN = 80 : 20 to 40 : 60 over 60 min at 2.5 mL/min).
